# The radiosensitizing effects of a STAT3/HDAC dual-target inhibitor derived from isoalantolactone in solid tumor models

**DOI:** 10.1186/s12885-026-15816-7

**Published:** 2026-03-06

**Authors:** Qi Wang, Haiyan Qiu, Rongfei Zhu, Rong Li, Jiahan Qiao, Chun Fu, Yaxin Tang, Aihua Shen, Junfang Yan, Denggao Zhao, Burong Hu

**Affiliations:** 1 School of Public Health, Wenzhou Medical University, Wenzhou, Zhejiang 325035 China; 2 Shenzhen Hospital, Beijing University of Chinese Medicine, Shenzhen, Guangdong 518100 China; 3 School of Pharmacy and Food Engineering, Wuyi University, Jiangmen, 529020 China; 4 Zhejiang Engineering Research Center for Innovation and Application of Intelligent Radiotherapy Technology, The Second Affiliated Hospital of Wenzhou Medical University, Wenzhou, Zhejiang 325000 China

**Keywords:** STAT3/HDAC, Isoalantolactone, Dual-target inhibitor, Radiosensitization, Antitumor

## Abstract

Multi-target combination therapy has emerged as a promising anti-cancer strategy. STAT3 and HDAC are key contributors to tumorigenesis and progression. We previously reported the design of a novel dual-target inhibitor of STAT3 and HDAC (named mhl-28), which was developed by employing a naturally occurring STAT3 inhibitor derived from the traditional medicinal herb Saussurea as the pharmacophore and incorporating an HDAC- inhibitory structural motif. Here, mhl-28 was shown to simultaneously inhibit STAT3 and HDAC activities, demonstrating remarkable anti-proliferation and radiosensitizing effects against various solid cancer cell lines. In vitro, mhl-28 significantly suppresses proliferation and colony formation across various solid cancer cell lines. When combined with radiotherapy, it enhanced DNA damage and micronucleus formation, increased oxidative stress and apoptosis, induced cell cycle arrest, and inhibited tumor cell migration. In vivo experiments, mhl-28 combined with radiotherapy leads to significantly greater suppression subcutaneous tumor growth in mice, demonstrating more potent antitumor and radiosensitizing effects than SAHA. These findings indicate that mhl-28, a dual-target agent derived from traditional medicine, has strong potential as an anti-tumor drug in solid tumor therapy when combined with radiation.

**Supplementary Information:**

The online version contains supplementary material available at 10.1186/s12885-026-15816-7.

## Introduction

The complexity of cancer progression, driven by a highly intricate network of molecular and cellular interactions, has underscored the limitations of targeting single molecules or pathways in cancer therapy. Multi-target combination therapy has emerged as a promising strategy that simultaneously addresses multiple mechanisms underlying tumor growth, survival, and resistance [1, 2]. By modulating several oncogenic pathways concurrently, multi-target agents not only enhance therapeutic efficacy but also reduce the likelihood of drug resistance- a major limitation of conventional monotherapies. In the context of solid tumors, where resistance to single-target agents frequently compromise treatment outcomes, dual-targeted inhibitors provide a rational and effective approach to overcoming these challenges.

Traditional Chinese Medicine (TCM) and other natural medicinal sources have shown considerable promise in cancer treatment. A key strength of TCM lies in its rich reservoir of bioactive compounds, which often exert multi-target effects and modulate multiple cancer-related signaling pathways. In recent decades, phytochemicals derived from traditional herbs have attracted growing attention due to their roles in both primary and adjunctive cancer therapies. Natural compounds such as STAT3 (Signal Transducer and Activator of Transcription 3) and HDAC (Histone Deacetylase) inhibitors offer significant advantages, including reduced side effects and enhanced therapeutic efficacy through synergistic interactions with conventional drugs or modalities such as radiotherapy. As a result, TCM-derived agents have emerged as promising candidates for drug development, meeting the increasing demand for novel, multi-targeted strategies in oncology.

Among the emerging therapeutic targets, STAT3 [3, 4] and HDAC have garnered significant interest in cancer treatment due to their critical roles in tumor growth, survival, and metastasis. STAT3 is frequently overactivated in various cancers and regulates genes involved in cell proliferation, apoptosis evasion, and immune suppression, Its inhibition has shown potential in slowing tumor progression [5, 6]. Isoalantolactone (IAL), a bioactive compound derived from Saussurea costus, act as a natural STAT3 inhibitor by selectively promoting STAT3 glutathionylation, thereby blocking phosphorylation and subsequent nuclear translocation, ultimately suppressing cancer cell growth [7–9]. In parallel, HDAC inhibitors (HDACi), known for their ability to modulate gene expression through epigenetic mechanisms by altering chromatin structure [10–12], have shown promise in treating hematological malignancies [13–22]. However, their efficacy against solid tumors remains limited [20, 23–30]. Notably, recent studies have revealed that HDAC inhibition in certain cancer models can inadvertently activate the JAK-STAT3 signaling pathway, potentially counteracting the intended anti-tumor effects [31, 32]. This compensatory activation highlights a key mechanism of resistance and has prompted investigation into novel combinatorial strategies that simultaneously target HDAC and the BRD4-LIFR-JAK-STAT3 pathway to overcome resistance and enhance therapeutic efficacy [33–35].

Radiotherapy (RT) is a cornerstone in the treatment of solid tumors, leveraging precise targeting of tumor cells to induce DNA damage and inhibit cancer cell proliferation. However, radioresistance remains a major clinical challenge, significantly limiting the effectiveness of RT across various cancer types. Given the established roles of STAT3 and HDAC in regulating DNA damage response and cellular repair pathways, dual inhibition of these targets represents a promising strategy to enhance radiosensitivity. Building on this rationale, we developed mhl-28 (formerly compound 18), a novel STAT3-HDAC dual-target inhibitor derived from IAL—a natural medicinal compound—integrated with the pharmacophoric moiety of an HDAC inhibitor, with the aim of improving therapeutic efficacy and overcoming limitations associated with conventional HDAC inhibitors in solid tumors [36]. Our primary studies have demonstrated that mhl-28 exhibits potent antitumor activity against A549 xenograft tumors [36]. In this study, we systematically evaluate the dual inhibitory effects of mhl-28 on tumor growth and its ability to sensitize tumors to radiation, with direct comparison to the canonical HDAC inhibitor SAHA (Vorinostat). Furthermore, the antitumor efficacy of mhl-28 was assessed in B16 melanoma xenograft models. These findings underscore the potential of mhl-28 as a naturally derived therapeutic agent capable of enhancing radiotherapy efficacy through targeted molecular modulation, thereby providing novel strategic approaches for oncology combination therapies.

## Result

### mhl-28 combined with IR (Ionization Radiation) inhibits STAT3 and HDAC activity in tumor cells

**Fig. 1:**
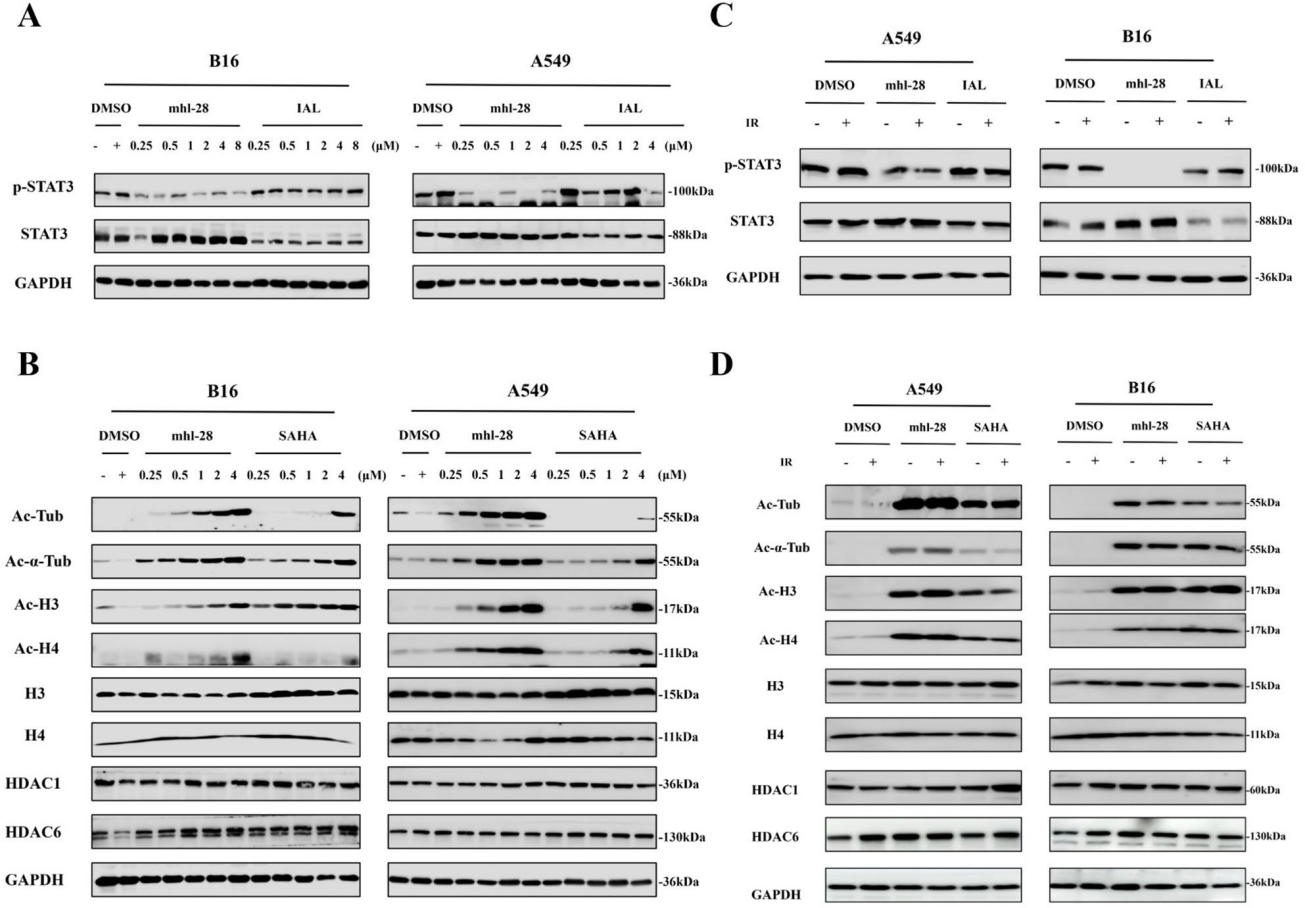
mhl-28 combined with IR inhibits STAT3 and HDAC activity in tumor cells. **A** Protein expression levels of p-STAT3 (Tyr705) and STAT3 were assessed in A549 and B16 cells following treatment with 0.25, 0.5, 1, 2, 4, 8 µM of mhl-28 or IAL (isoalantolactone) for 24 h. **B** Protein expression levels of Ac-Tub, Ac-α-Tub, Ac-H3, Ac-H4, H3, H4, HDAC1 and HDAC6 were assessed in A549 and B16 cells following treatment with 0.25, 0.5, 1, 2, 4 µM of mhl-28 or SAHA for 24 h. **C** Protein expression levels of p-STAT3 (Tyr705) and STAT3 were assessed in A549 and B16 cells following treatment with 0.5 µM of mhl-28 or IAL for 24 h, after which the cells were exposed to 4 Gy of X-rays. **D** Protein expression levels of Ac-Tub, Ac-α-Tub, Ac-H3, Ac-H4, H3, H4, HDAC1 and HDAC6 were assessed in A549 and B16 cells following treatment with 0.5 µM of mhl-28 or SAHA for 24 h, after which the cells were exposed to 4 Gy of X-rays. DMSO (-) represents the blank control group (untreated), while DMSO (+) denotes the 0.1% DMSO vehicle control group. Data are presented as mean ± SD from three independent experiments (*n* = 3)

As a rationally designed dual-target inhibitor, our results demonstrate that mhl-28 effectively suppresses the activation of STAT3, as evidenced by reduced phosphorylation levels (STAT3 Tyr7055), whereas IAL at equivalent concentrations exerts only a marginal effect on STAT3 phosphorylation (Fig. 1A & Extended Fig. 1). Furthermore, compared to SAHA at the same concentration, treatment with mhl-28 leads to a significant increase in the acetylation levels of α-tubulin, histone H3, and histone H4, indicating potent inhibition of HDAC1 and HDAC6 enzymatic activity (Fig. 1B & Extended Fig. 2). Notably, similar effects are observed when mhl-28 is combined with 4 Gy radiation, with no substantial difference compared to mhl-28 alone (Fig. 1C-D & Extended Figs. 3 and 4), suggesting that the compound maintains its inhibitory activity under irradiation conditions. These findings support a potential synergistic radiosensitizing effect of mhl-28 in combination with radiation therapy.

### mhl-28 inhibits tumor cell proliferation in vitro

Given the primary objective of developing a dual-target inhibitor for various solid tumors, we evaluated the effects of mhl-28 on human non-small cell lung cancer (A549), human breast cancer (MDA-MB-231), and murine melanoma (B16) cells, with the latter serving as a well-established model of refractory tumor in mice for preclinical studies, by exposing these cells to increasing concentrations of the compound. The CCK-8 assay revealed a consistent, dose-dependent reduction in cell viability with escalating mhl-28 concentrations, indicating significant inhibition of tumor cell proliferation (Extended Fig. 5).

**Fig. 2:**
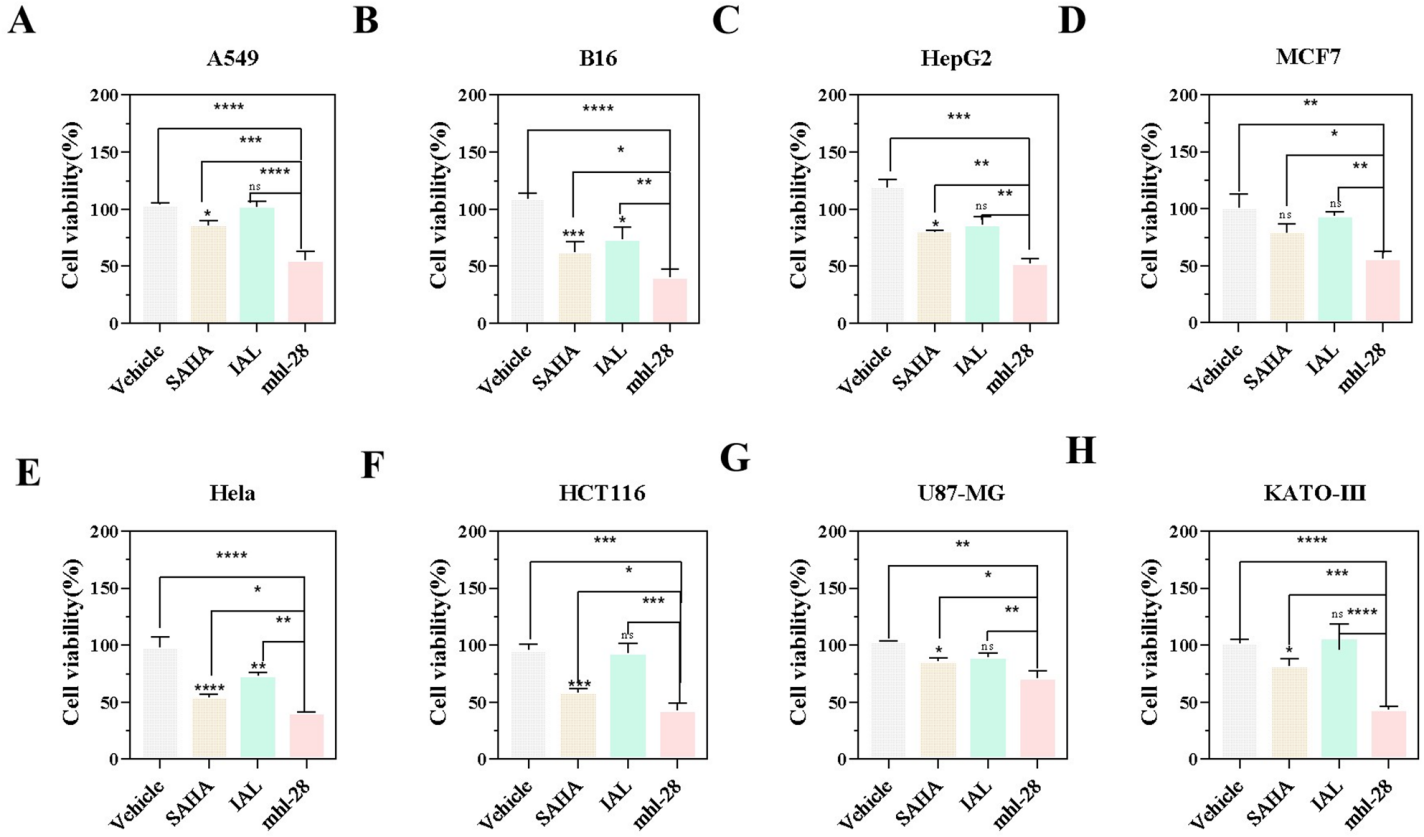
mhl-28 demonstrates enhanced proliferative inhibition on various solid tumor cell lines. A549 (**A**), B16 (**B**), HepG2 (**C**), MCF7 (**D**), Hela (**E**), HCT116 (**F**), U87-MG (**G**), and KATO-III (**H**) cells were treated with 0.5 µM mhl-28, SAHA, and IAL for 48 h, followed by cell viability assessment using the CCK-8 assay. Data are presented as mean ± SD from three independent experiments (*n* = 3). The vehicle control group was treated with 0.1% DMSO. Statistical significance: **Asterisks (*, **, ***, **) above bars indicate comparisons with the Vehicle group. Asterisks above horizontal lines indicate comparisons with the mhl-28 group. “ns” indicates no statistical significance. *P < 0.05, **P < 0.01, ***P < 0.001, ****P < 0.0001

Next, the anti-tumor activity of mhl-28 was evaluated in vitro across various solid tumor cell lines, including human cancer cell lines (A549, HepG2, MCF-7, HeLa, HCT16, U87-MG, and KATO-III) and murine melanoma cells (B16), and compared with that of SAHA and IAL as reference (positive) controls using the standard CCK-8 assay. In all tested solid tumor cell lines, mhl-28 demonstrated superior anti-proliferative activity relative to both SAHA and IAL at equivalent concentrations (Fig. 2). At 0.5 µM, IAL exerted only a marginal effect on most cancer cell lines, while SAHA exhibited moderate anti-proliferative activity. In contrast, mhl-28 consistently and significantly suppressed tumor cell growth. Therefore, SAHA was selected as the positive control in subsequent experiments. Overall, mhl-28 displayed greater potency and sensitivity compared to both reference compounds.

### mhl-28 combined with IR inhibits clonogenic capacity

The colony formation assay is a widely recognized and highly effective method for evaluating the potential of compounds to suppress cellular clone formation and tumorigenic capacity. We employed this assay to assess whether mhl-28 possesses such inhibitory effects. With increasing concentrations of mhl-28, the survival fraction of A549 cells decreased significantly (Extended Fig. 6), indicating that mhl-28 effectively suppresses clonogenic activity in A549 cells in a dose-dependent manner. Notably, mhl-28 demonstrated greater inhibitory potency compared to SAHA at equivalent doses. These results are consistent with the findings from the CCK-8 assay, further supporting its anti-proliferative efficacy.

**Fig. 3:**
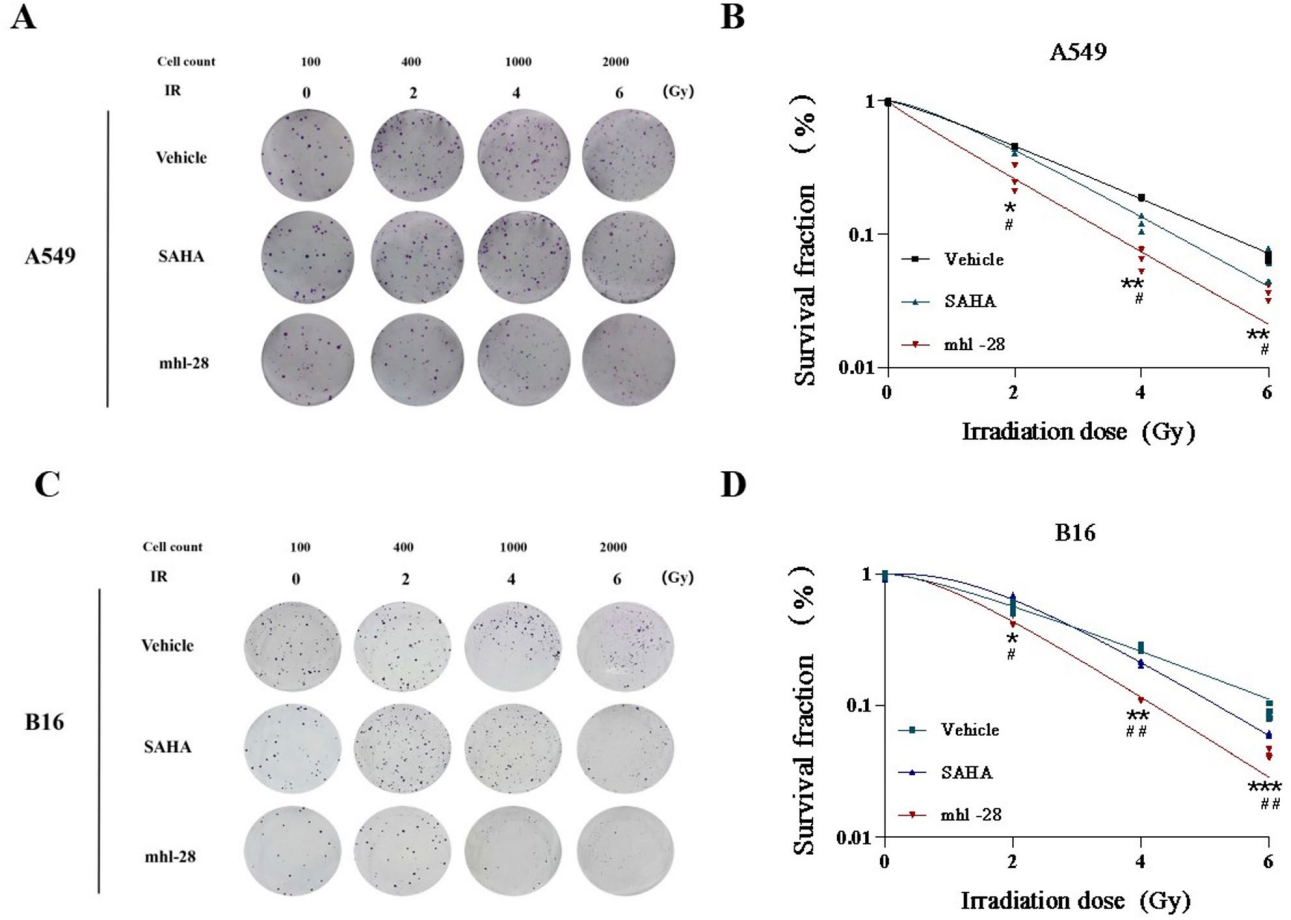
mhl-28 combined with IR inhibits the clonogenic capacity of tumor cells. Representative images of clonogenic survival of A549 cells (**A**) and B16 cells (**C**) treated with 0.5 µM mhl-28 or SAHA for 24 h, followed by exposure to 0, 2, 4, or 6 Gy of X-rays; quantified clonogenic survival fractions modeled using the single-hit multi-target model (**B**, **D**). The vehicle control group was treated with 0.1% DMSO. Data are presented as mean ± SD from three independent experiments (*n* = 3). Statistical significance: ‘ns’ indicates no statistical significance, * *P* < 0.05, ** *P* < 0.01, *** *P* < 0.001, and **** *P* < 0.0001. * indicates statistical significance between the vehicle control group and the mhl-28 group at each dose level. Statistical significance: ^#^ *P* < 0.05, ^##^ *P* < 0.01, ^###^ *P* < 0.001, and ^####^ *P* < 0.0001. ^#^ indicates statistical significance between the SAHA and mhl-28 groups at each dose level

The colony formation assay, as well as regarded as the gold standard for assessing radiosensitivity, evaluates cancer cells’ resistance to radiation by quantifying the number and size of colonies formed by individual cells following irradiation. In this study, we investigated the radiosensitizing effects of combining SAHA or mhl-28 with irradiation on A549 and B16 cells, generating clonogenic survival curves based on the experimental results. Compared to both the control and SAHA treatment groups, mhl-28 treatment resulted in significantly reduced clonogenic survival in both A549 and B16 cells, demonstrating a clear dose-dependent response to radiation (Fig. 3), indicating enhanced radiosensitivity relative to the control and SAHA.

### mhl-28 combined with IR promotes DNA damage and micronucleus formation

**Fig. 4:**
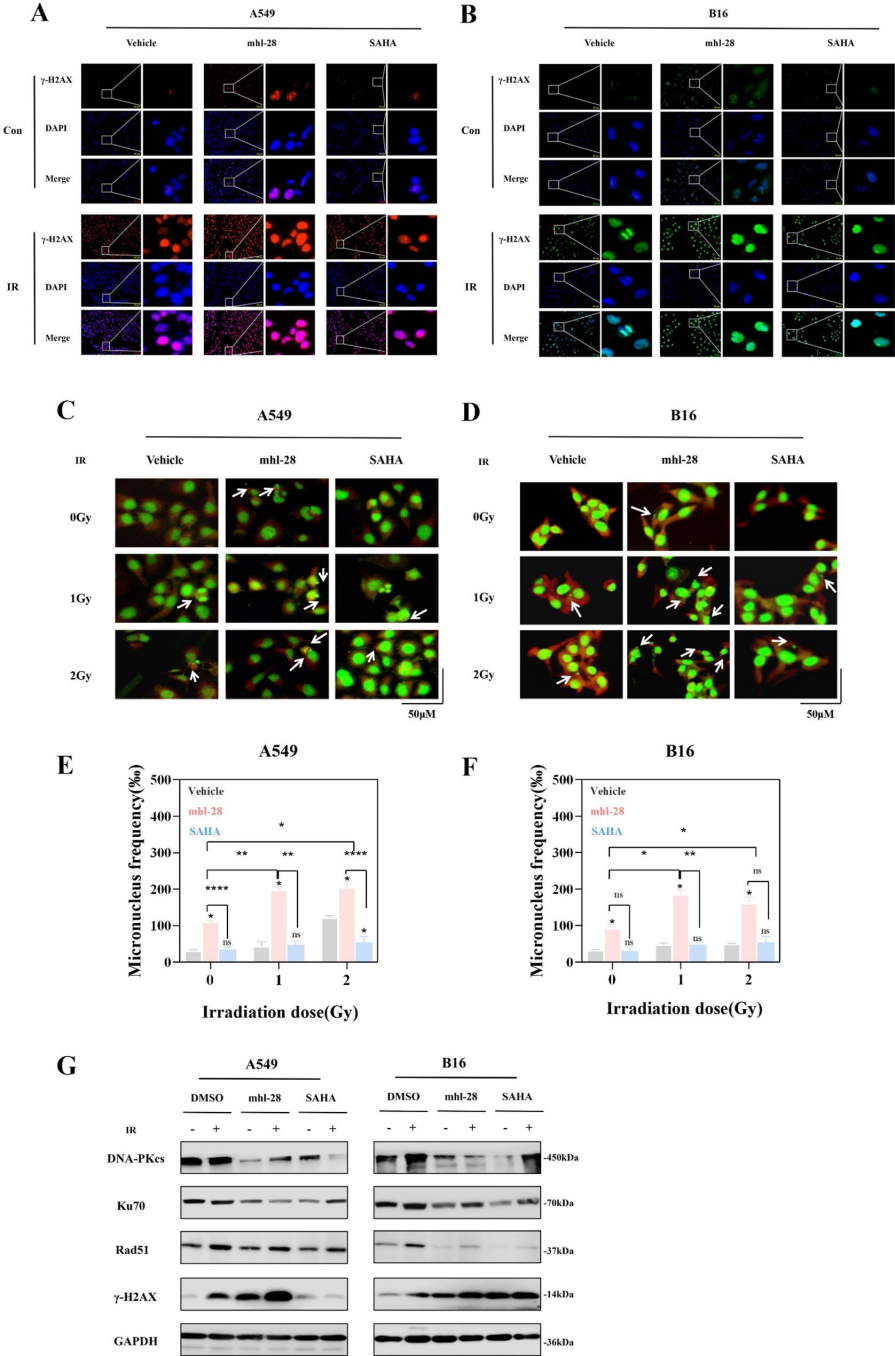
mhl-28 combined with IR exacerbates DNA damage and micronuclei formation in tumor cells. **A**, **B** Representative immunofluorescence images of γ-H2AX foci in A549 (**A**) and B16 (**B**) cells treated with 0.5 µM mhl-28 for 0.5 h, followed by exposure to 4 Gy of X-rays. **C**, **D** Represen tative images of micronucleus formation in A549 (**C**) and B16 (**D**) cells treated with 0.5 µM mhl-28 for 24 h, followed by exposure to 0, 1, or 2 Gy of X-rays and assessed 24 h later. (E, F) Quantification of micronucleus frequency in A549 (**C**) and B16 (**D**) cells. **G** Protein expression levels of DNA-PKcs, Ku70, Rad51 and γ-H2AX were assessed in A549 and B16 cells following treatment with 0.5 µM of mhl-28 or SAHA for 24 h, after which the cells were exposed to 4 Gy of X-rays. The vehicle control group was treated with 0.1% DMSO. Data are presented as mean ± SD from three independent experiments (*n* = 3). Statistical significance: Asterisks above bars (*, **, ***, ****) indicate comparisons with the irradiation-only control group at the same dose. Asterisks above horizontal lines (*, **, ***, ****) indicate pairwise comparisons within the bracketed groups. ‘ns’ indicates no statistical significance. * *P* < 0.05, ** *P* < 0.01, *** *P* < 0.001, **** *P* < 0.0001

Multiple studies have demonstrated that dual inhibition of HDAC and STAT3 disrupts DNA double-strand break (DSB) repair pathways and enhances radiosensitivity. To determine whether mhl-28 in combination with IR impairs DNA damage repair, we assessed γ-H2AX foci formation—a widely recognized marker of DSBs that reflects phosphorylation of histone H2AX at damage sites within minutes after irradiation. Immunofluorescence analysis in A549 and B16 cells showed significantly higher numbers of γ-H2AX foci 0.5 h after exposure to 4 Gy IR in the mhl-28 pretreated groups compared to both SAHA-treated and vehicle controls (Fig. 4A and B). The persistence of γ-H2AX foci suggests impaired activation of DNA repair mechanisms.

The micronucleus assay is an in vitro cytogenetic method used to assess drug-induced chromosomal damage (genotoxicity). To further investigate the effect of mhl-28 in combination with IR on chromosomal stability—specifically, radiosensitivity—we employed acridine orange staining to evaluate micronucleus formation in A549 and B16 cells following X-ray irradiation after drug treatment. As shown in Fig. 4C–F, the combination of mhl-28 and IR significantly increased micronucleus formation compared to controls. Complementary western blot analysis of core DNA repair proteins revealed that the mhl-28/IR combination markedly altered key DNA repair pathways (Fig. 4G & Extended Fig. 7). Notably, mhl-28 enhanced IR-induced DNA damage more effectively than equimolar SAHA, indicating a superior ability to impair both major DSB repair mechanisms: DNA-PKcs/Ku70-mediated non-homologous end joining (NHEJ), an error-prone pathway, and Rad51-dependent homologous recombination (HR), which enables high-fidelity repair [37].

### mhl-28 combined with IR inhibits A549 cell migration

Cell migration is a critical step in cancer progression, and accumulating evidence indicates that STAT3 overactivation serves as a key driver of this process. Moreover, α-tubulin, a downstream substrate of HDAC6, constitutes a fundamental component of the cytoskeleton and subcellular architectures, playing an essential role in tumor cell motility and showing a strong correlation with the risk of metastasis and recurrence [38, 39]. Therefore, mhl-28 may modulate tumor metastasis by regulating STAT3 activation and the acetylation status of α-tubulin, thereby influencing tumor cell motility. To test this hypothesis, we evaluated the anti-migratory effect of mhl-28. At a concentration of 0.5 µM, mhl-28 significantly suppressed A549 cell migration (Extended Fig. 8A and B). Notably, the combination of 0.5 µM mhl-28 with X-ray irradiation led to a greater reduction in migratory capacity compared to mhl-28 treatment alone (Extended Fig. 8C and D).

**Fig. 5:**
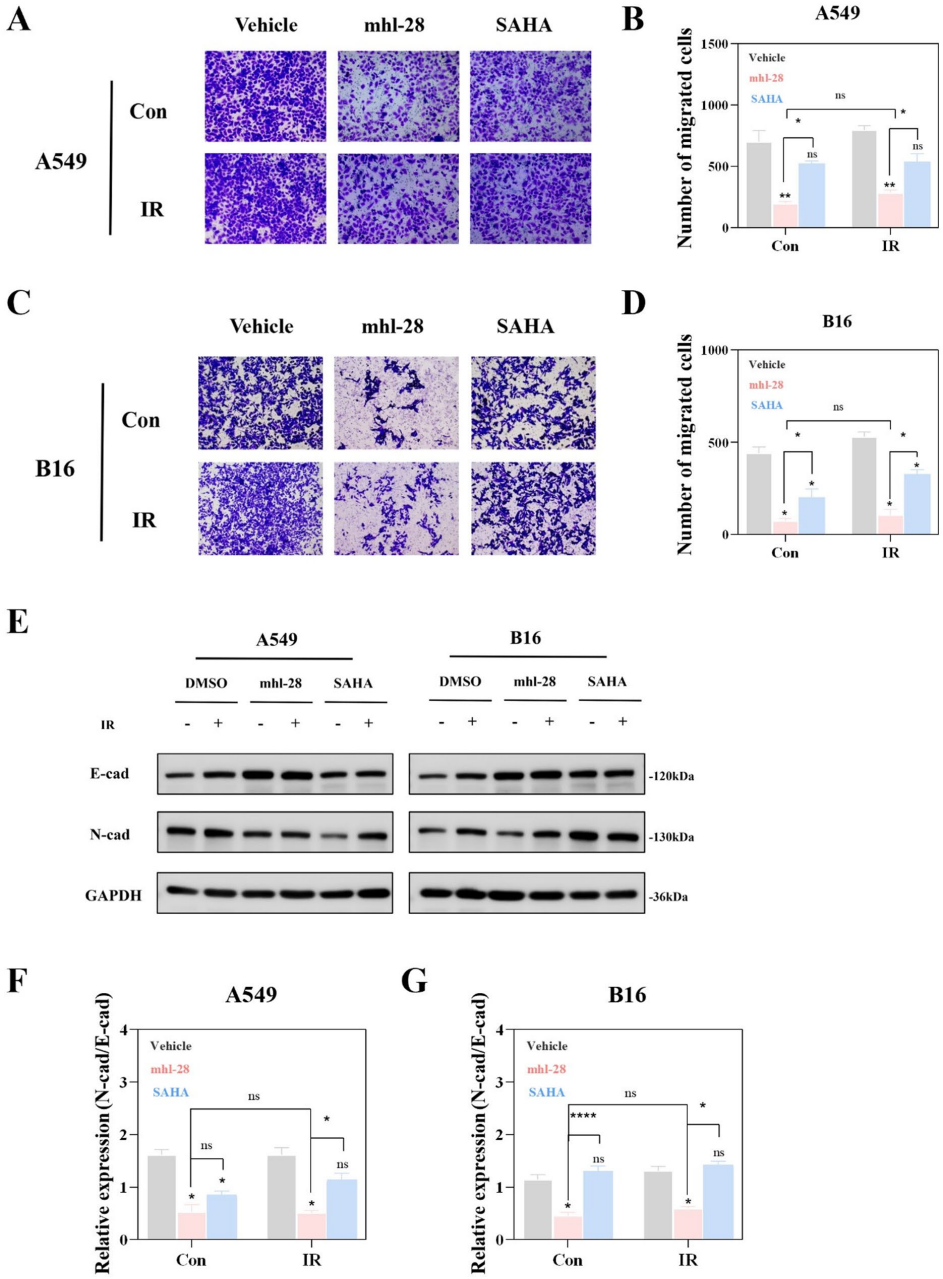
mhl-28 combined with IR inhibits migration in tumor cells. **A**, **B** Transwell migration assay of A549 (**A**) and B16 (**B**) cells following mhl-28 or SAHA treatment combined with irradiation. Cells were pretreated with 0.5 µM mhl-28 or SAHA for 24 h before exposure to X-ray radiation (4 Gy). Representative images show migrated cells on the lower membrane surface after 24 h post-irradiation (Crystal violet staining; scale bar: 100 μm). **C**, **D** Quantification of migration cells in A549 (**C**) and B16 (**D**) cells. **E** Protein expression levels of E-cad and N-cad were assessed in A549 and B16 cells following treatment with 0.5 µM of mhl-28 or SAHA for 24 h, after which the cells were exposed to 4 Gy of X-rays. **F**, **G** Quantitative ratio of N-cadherin to E-cadherin (N-cad/E-cad) protein expression in A549 (**F**) and B16 (**G**) cells. The vehicle control group was treated with 0.1% DMSO. Data are presented as mean ± SD from three independent experiments (*n* = 3). Statistical significance: Asterisks above bars (*, **, ***, ****) indicate comparisons with the irradiation-only control group at the same dose. Asterisks above horizontal lines (*, **, ***, ****) indicate pairwise comparisons within the bracketed groups. ‘ns’ indicates no statistical significance. * *P* < 0.05, ** *P* < 0.01, *** *P* < 0.001, **** *P* < 0.0001

The anti-migratory effect of mhl-28 was further evaluated using Transwell migration assays, which revealed that mhl-28 treatment significantly inhibited the migration of A549 and B16 cells following radiation, compared to the SAHA group at the same concentration (Fig. 5A–D). Moreover, the decreased N-cadherin/E-cadherin ratio, a marker associated with enhanced metastatic potential, provided molecular evidence supporting the observed suppression of cell migration [40]. Western blot analysis confirmed that mhl-28 treatment upregulated N-cadherin expression while downregulating E-cadherin, consistent with inhibition of tumor cell migratory capacity (Fig. 5E–G).

### mhl-28 combined with IR promotes apoptosis

**Fig. 6:**
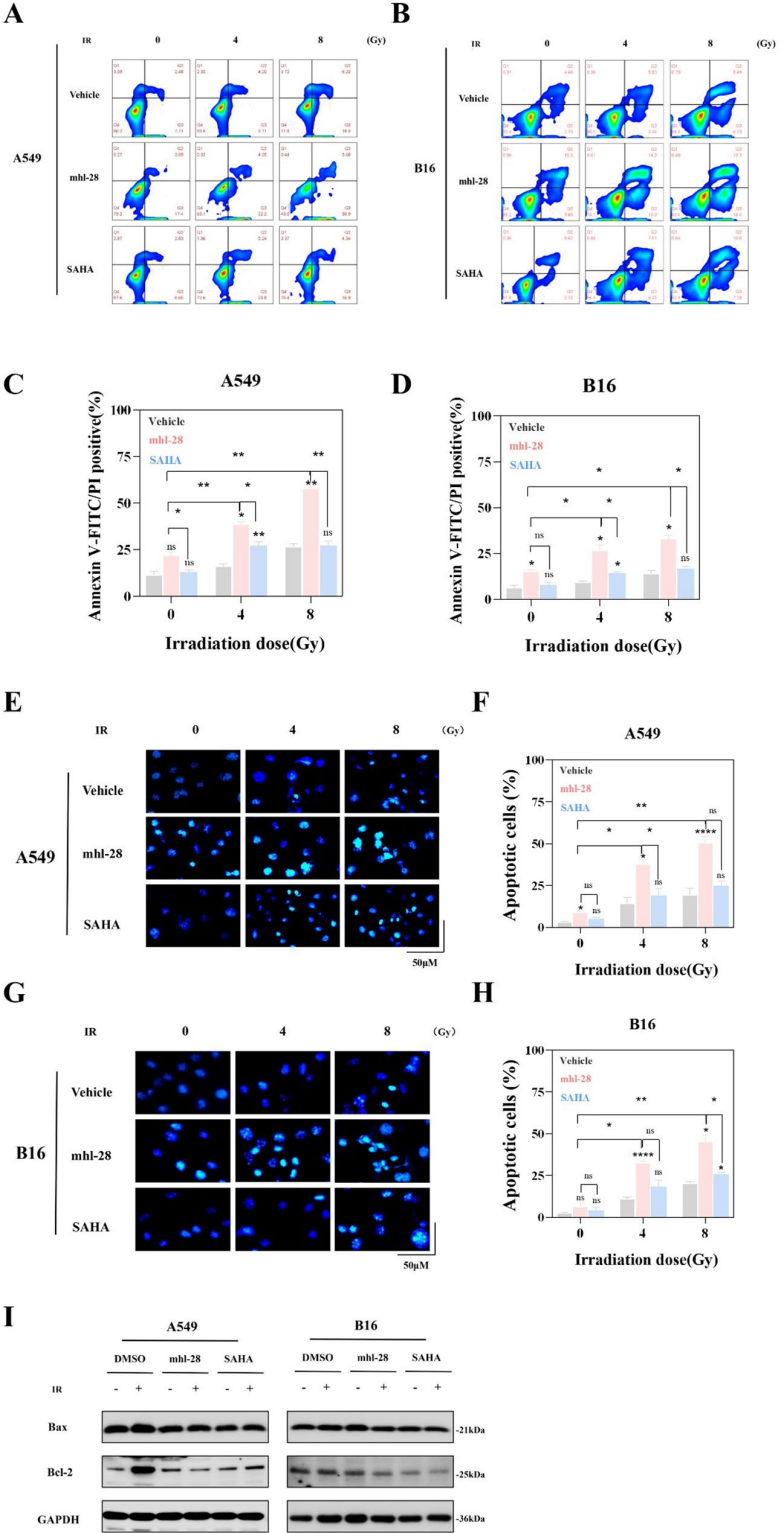
mhl-28 combined with IR promotes apoptosis in tumor cells. **A**-**D** Representative flow cytometry images and quantified apoptosis rates of A549 cells (**A** & **C**) and B16 cells (**B** & **D**) treated with 0.5 µM mhl-28 or SAHA for 24 h, followed by exposure to 0, 4, or 8 Gy of X-rays, assessed 24 h later. **E**-**H** Representative images and quantified apoptosis rates of A549 cells (**E** & **F**) and B16 cells (**G** & **H**) stained with Hoechst 33,342 after treatment with 0.5 µM mhl-28 or SAHA for 24 h and exposure to 0, 4, or 8 Gy of X-rays, assessed 24 h later. **I** Protein expression levels of Bax and Bcl-2 were assessed in A549 and B16 cells following treatment with 0.5 µM of mhl-28 or SAHA for 24 h, after which the cells were exposed to 4 Gy of X-rays. The vehicle control group was treated with 0.1% DMSO. Data are presented as mean ± SD from three independent experiments (*n* = 3). Statistical significance: Asterisks above bars (*, **, ***, ****) indicate comparisons with the irradiation-only control group at the same dose. Asterisks above horizontal lines (*, **, ***, ****) indicate pairwise comparisons within the bracketed groups. ‘ns’ indicates no statistical significance. * *P* < 0.05, ** *P* < 0.01, *** *P* < 0.001, **** *P* < 0.0001

Inhibiting HDACs and/or STAT3 is known to induce apoptosis in cancer cells. We evaluated the effects of mhl-28 on apoptosis using three complementary approaches. First, Annexin V/PI staining followed by flow cytometric analysis was employed to quantify apoptosis rates in cells treated with mhl-28 in combination with radiation therapy (0, 4, or 8 Gy). The proportion of apoptotic cells in mhl-28-treated groups was significantly higher than that in both SAHA-treated and control groups (Fig. 6A–D). Notably, mhl-28-induced apoptosis in A549 and B16 cells exhibited a radiation dose-dependent increase, reaching 63% in A549 cells and 42% in B16 cells at 8 Gy. Second, A549 cells pretreated with varying concentrations of mhl-28 were stained with Hoechst 33,342 and examined under a fluorescence microscope. Characteristic nuclear condensation, indicative of apoptosis, was observed in mhl-28-treated cells (Fig. 6E–H). Furthermore, western blot analysis of key apoptosis-related proteins yielded consistent results (Fig. 6I & Extended Fig. 9). Collectively, these findings demonstrate that mhl-28, when combined with irradiation, effectively promotes apoptosis in tumor cells.

### mhl-28 combined with IR potentiates radiation-induced oxidative stress

**Fig. 7:**
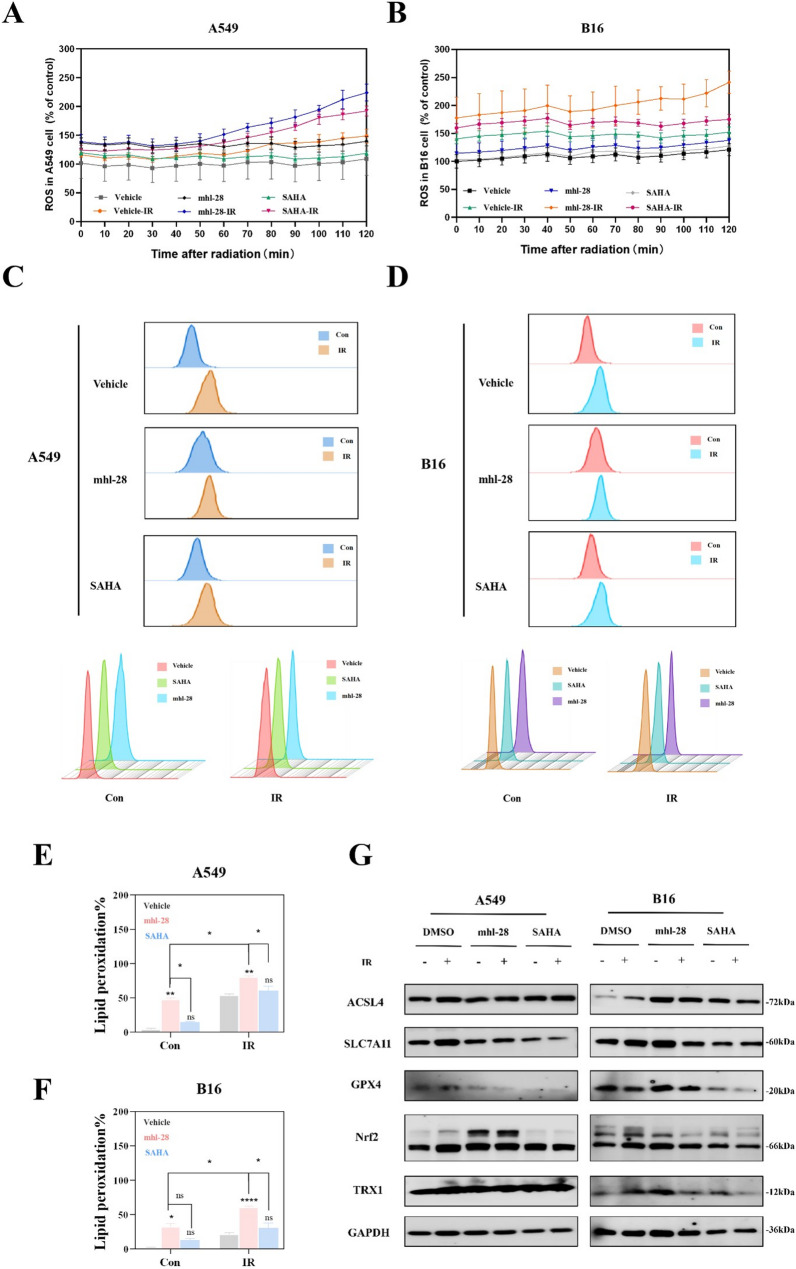
mhl-28 combined with IR induces oxidative stress in tumor cells. **A**, **B** Kinetic profiles of ROS levels in A549 (**A**) and B16 (**B**) cells post-irradiation. Cells pretreated with 0.5 µM mhl-28 or SAHA for 24 h were exposed to 4 Gy X-rays. Intracellular ROS was measured by DCFH-DA fluorescence at 0–120 min post-irradiation. **C**-**F** Representative flow cytometry images and quantified lipid peroxidation rates of A549 cells (**C** & **E**) and B16 cells (**D** & **F**) treated with 0.5 µM mhl-28 or SAHA for 24 h, followed by exposure to 10 Gy of X-rays, assessed 48 h later. Lipid peroxidation detection by flow cytometry 48 h post-irradiation. Representative histogram plots of A549 and B16 cells stained with C11-BODIPY⁵⁸¹/⁵⁹¹. **G** Protein expression levels of ACSL4, SLC7A11, GPX4, Nrf2 and TRX1 were assessed in A549 and B16 cells following treatment with 0.5 µM of mhl-28 or SAHA for 24 h, after which the cells were exposed to 4 Gy of X-rays. The vehicle control group was treated with 0.1% DMSO. Data are presented as mean ± SD from three independent experiments (*n* = 3). Statistical significance: Asterisks above bars (*, **, ***, ****) indicate comparisons with the irradiation-only control group at the same dose. Asterisks above horizontal lines (*, **, ***, ****) indicate pairwise comparisons within the bracketed groups. ‘ns’ indicates no statistical significance. * *P* < 0.05, ** *P* < 0.01, *** *P* < 0.001, **** *P* < 0.0001

Ionizing radiation directly damages DNA while simultaneously generating secondary reactive oxygen species (ROS) that propagate genomic instability [41]. To evaluate redox imbalance following combined treatment, intracellular ROS levels were measured using DCFH-DA fluorescence. In A549 and B16 cells exposed to 4 Gy irradiation, mhl-28-treated resulted in greater ROS accumulation than equimolar SAHA from 0 to 120 min post-irradiation (Fig. 7A & B), indicating enhanced potentiation of oxidative stress.

Lipid peroxidation, a hallmark of ferroptotic cell death, was evaluated by flow cytometry using C11-BODIPY^581/591^, a fluorescent probe that shifts from red to green fluorescence upon oxidation by ROS [42]. The mhl-28/IR combination induced significantly greater accumulation of lipid peroxidation compared to SAHA/IR in both cell lines **(**Fig. 7C–F), indicating a superior capacity to promote membrane oxidative damage. Western blot analysis of redox-regulatory proteins further demonstrated that mhl-28 exerts a multifaceted influence on cellular oxidative homeostasis (Fig. 7G & Extended Fig. 10). Collectively, these findings establish that mhl-28 generates a sustained oxidative stress signature in irradiated tumor cells, disrupting redox equilibrium more effectively than SAHA at equivalent concentrations.

### mhl-28 combined with IR induces G2/M cell cycle arrest

**Fig. 8:**
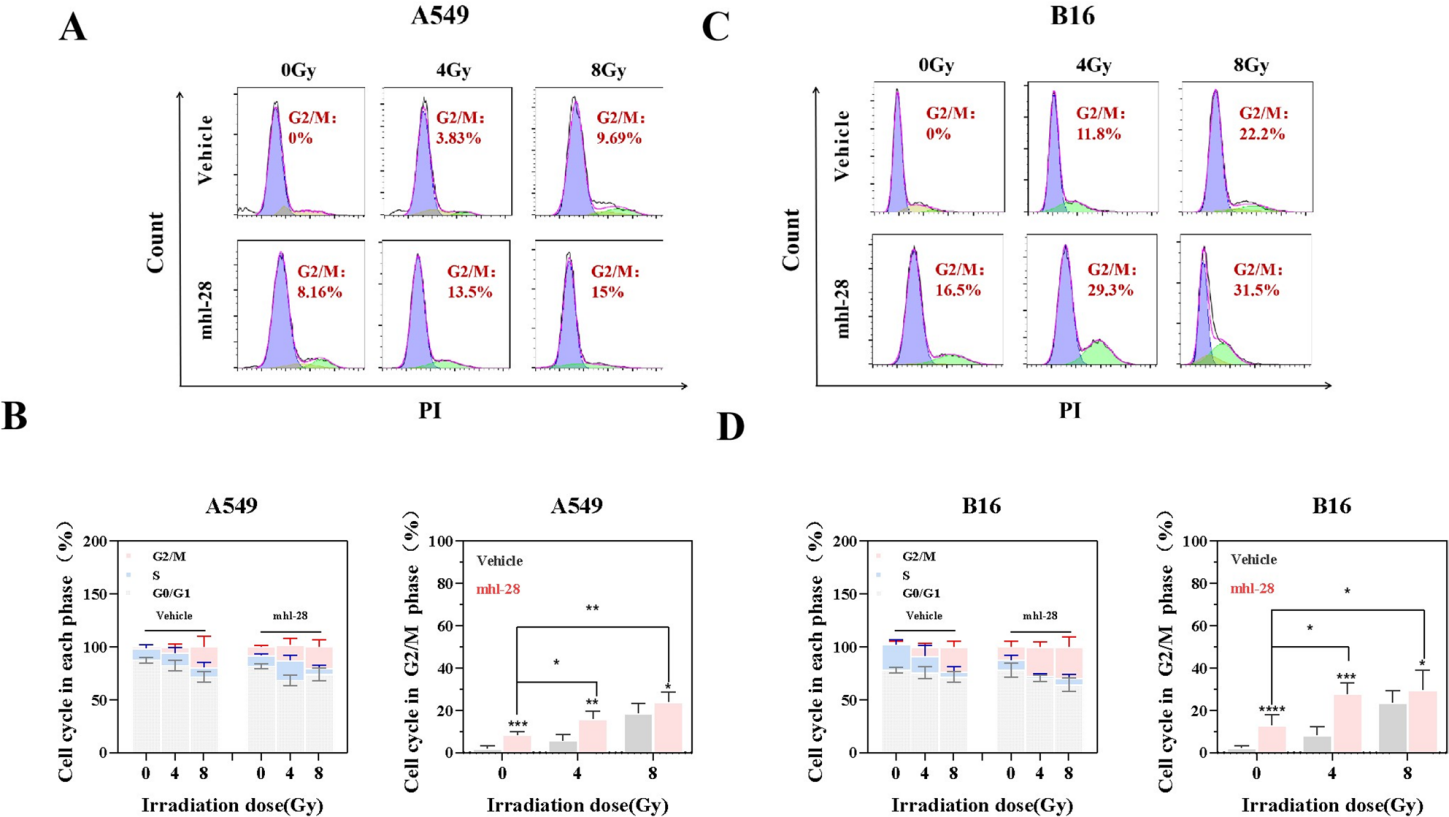
mhl-28 combined with IR induces G2/M phase cell cycle arrest. **A**, **C** Representative flow cytometry images of cell cycle distribution in A549 cells (**A**) and B16 cells (**C**) treated with 0.5 µM mhl-28 for 24 h, followed by exposure to 0, 4, or 8 Gy of X-rays, assessed 24 h later. **B**, **D** Quantified cell cycle distributions, focusing on the G2/M phase distribution in A549 cells (**B**) and B16 cells (**D**) after treatment. The vehicle control group was treated with 0.1% DMSO. Data are presented as mean ± SD from three independent experiments (*n* = 3). ‘ns’ indicates no statistical significance, * *P* < 0.05, ** *P* < 0.01, *** *P* < 0.001, and **** *P* < 0.0001

We next evaluated the impact of mhl-28 on cell cycle regulation. A549 and B16 cells were treated with mhl-28 for 24 h prior to exposure to receiving 0, 4, or 8 Gy radiation therapy. Cell cycle analysis revealed that, compared to DMSO control, mhl-28 significantly increased the proportion of cells in the G2/M phase in both A549 and B16 cells (Fig. 8). Since radiation is known to induce G2/M phase arrest in tumor cells, our findings indicate that mhl-28 enhances this radiation-induced cell cycle arrest, thereby potentiating its cytotoxic effects.

### mhl-28 combined with IR inhibits tumor growth in vivo

**Fig. 9:**
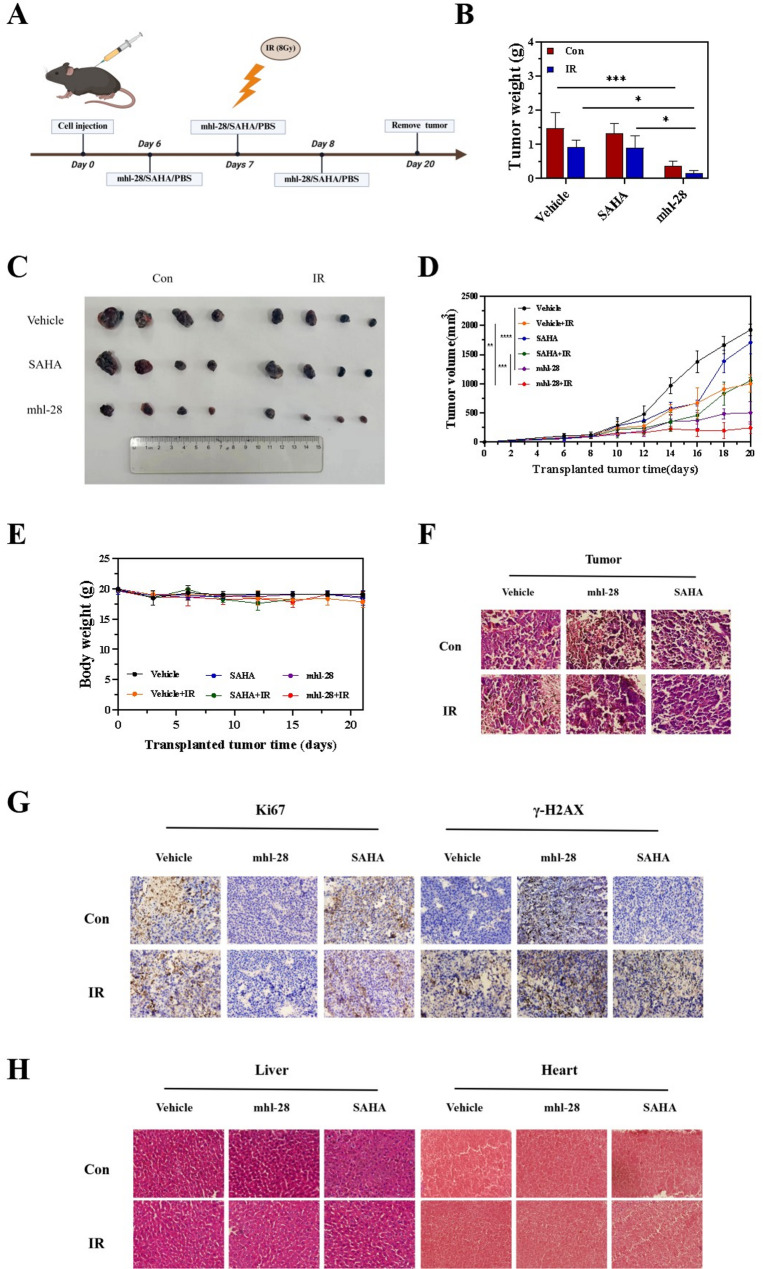
mhl-28 combined with IR inhibits tumor growth in vivo. **A** Scheme for RT combined with mhl-28 treatment. Mice were inoculated with B16 cells and randomly assigned on day 6 to receive intraperitoneal injections of equivalent doses of SAHA, mhl-28 and PBS on days 6, 7, and 8, with 8 Gy of irradiation on day 7. **B** Comparison of tumor weights on day 12 post-irradiation. **C** Images of tumors extracted from mice 12 days after IR. **D** Tumor growth curves in mice, showing delayed tumor growth in mhl-28 treated mice. **E** The body weight changes of different groups of mice. **F** H&E stain of analysis of tumor tissues of different groups of mice. **G** Typical immunohistochemical images of tumor tissues from different groups of mice (ki67 and γH2AX). (H) H&E staining of liver and heart of different groups of mice. The vehicle control group was injected with an equal volume of saline. ‘ns’ indicates no statistical significance, * *P* < 0.05, ** *P* < 0.01, *** *P* < 0.001, and **** *P* < 0.0001

After demonstrating promising anti-tumor activity in vitro, we evaluated the radiosensitizing effect of mhl-28 in vivo by establishing a subcutaneous melanoma model in C57BL/6 mice using B16 cells, a well-established murine model for studying refractory tumors. On day 6 post-inoculation, mice were randomly divided into six groups and received intraperitoneal injections of SAHA, mhl-28, or PBS on days 6, 7, and 8. The irradiated groups were exposed to 8 Gy of X-rays on day 7 (Fig. 9A). Both mhl-28 and radiation therapy—administered individually or in combination—reduced tumor volume. Notably, the group treated with mhl-28 combined with irradiation exhibited the lowest tumor weight (Fig. 9B). Tumor growth in the mhl-28 group was significantly slower compared to both the control and SAHA groups (Fig. 9C and D). Furthermore, the combination treatment resulted in significantly greater tumor growth inhibition than either monotherapy alone.

Meanwhile, no significant differences in body weight were observed across the treatment groups, suggesting that mhl-28 did not induce severe systemic toxicity (Fig. 9E). Furthermore, tumors treated with mhl-28 in combination with radiation exhibited increased necrosis, as shown by H&E staining (Fig. 9F), reduced tumor cell proliferation, as assessed by Ki67 immunohistochemistry, and elevated levels of DNA damage, as indicated by γ-H2AX immunohistochemical staining (Fig. 9G), compared to control and single-treatment groups. At the conclusion of the antitumor efficacy studies, H&E staining of major organs from all experimental groups revealed no notable pathological lesions, further supporting the favorable safety profile of mhl-28 **(**Fig. 9H**)**. To quantitatively assess the potential systemic toxicity of mhl-28, key serum biochemical markers of liver and kidney function were analyzed at the end of the treatment period. As shown in extended Fig. 11, compared to the PBS control group, neither mhl-28 monotherapy, IR alone, nor their combination caused any statistically significant elevation in serum levels of ALT, AST, CREA, or BUN (*p* > 0.05 for all comparisons). All measured values remained within the normal physiological ranges. Based on tumor growth delay analysis, the combination of IR and mhl-28 resulted in an enhanced antitumor effect.

## Discussion

In the development of novel oncology therapeutics, particularly in RT, where precise physical targeting of tumor cells is achievable, intrinsic tumor resistance to radiation remains a major limitation that significantly compromises therapeutic efficacy. Therefore, the development of agents capable of enhancing radiosensitivity—especially those that modulate multiple signaling pathways to improve treatment outcomes—is of critical importance. In this study, we report a novel dual-target inhibitor, mhl-28, which simultaneously inhibits HDAC and STAT3, demonstrating potent anti-proliferative activity and robust radiosensitizing effects in both in vitro and in vivo models.

HDAC and STAT3 play critical roles in regulating tumor growth, apoptosis, and differentiation. However, the underlying mechanisms contributing to the limited efficacy of HDAC inhibitors (HDACi) in solid tumors remain unclear. Previous studies have identified a feedback loop involving JAK1-STAT3 signaling that counteracts the therapeutic effects of conventional HDACi. Elevated STAT3 activity enhances anti-apoptotic signaling, which may explain the suboptimal response to HDACi monotherapy [32]. Notably, combined inhibition of HDAC and JAK has demonstrated synergistic effects in suppressing tumor cell proliferation, inducing apoptosis, and inhibiting tumor growth in heterotopic transplantation models [43, 44].

Compared to dual targeting of HDAC with either JAK1 or BRD4, simultaneous inhibition of the downstream effectors HDAC and STAT3 may represent a more effective therapeutic approach. Therefore, co-modulation of HDAC and STAT3 within feedback or compensatory activation pathways could help overcome tumor recurrence and drug resistance. In our study, mhl-28—a dual inhibitor of STAT3 and HDAC—overcomes the limitations associated with single-agent HDACi in solid tumors. Mechanistically, mhl-28 significantly reduces STAT3 phosphorylation (STAT3 Tyr7055) without altering total STAT3 protein levels, while increasing acetylation of histone H3 and α-tubulin. When combined with RT, mhl-28 enhances radiation-induced cell cycle arrest, DNA damage, and cell death. In vitro, mhl-28 increases micronucleus formation, suppresses cell migration, and potentiates radiation-induced apoptosis and oxidative stress. In vivo, the combination of mhl-28 and RT leads to a significant reduction in tumor growth in a murine melanoma model, highlighting its potential as a radiosensitizing agent.

Given the limited efficacy of HDAC inhibitors in solid tumors, we systematically assessed the antitumor activity of mhl-28 across a panel of solid tumor cell lines, including both human cancer cell lines and murine melanoma cells. Among the tested compounds, mhl-28 demonstrated superior inhibitory potency compared to SAHA and IAL, exhibiting the strongest antitumor effects. This enhanced efficacy is likely attributable to its dual inhibition of HDAC and STAT3 signaling pathways.

RT, which can target solid tumor cells with relative precision, is widely utilized in the treatment of most cancers. The efficacy of RT hinges on inducing DNA damage, thereby obstructing the replication and division of cancer cells. Studies have demonstrated that HDACi enhance radiosensitivity by sustaining elevated levels of histone acetylation, which in turn suppresses the recruitment and expression of key DNA damage repair factors, leading to prolonged repair of radiation-induced lesions [12, 45]. Moreover, signal transducer and activator of transcription 3 (STAT3) has been identified as a transcriptional regulator of XRCC1, a critical DNA repair factor involved in nucleotide excision repair and single-strand break (SSB) repair. XRCC1 is recruited to DNA damage sites in a poly(ADP-ribose) polymerase (PARP)-dependent manner, where it promotes genomic stability, inhibits apoptosis, and contributes to radiation resistance [9, 46]. Notably, the combination of RT and STAT3 inhibition results in significantly greater cancer cell death compared to either monotherapy alone [35]. Collectively, accumulating evidence indicates that dual inhibition of HDAC and STAT3 disrupts DSB repair pathways and potentiates the radiosensitizing effects of RT. As a novel dual-target inhibitor of both STAT3 and HDAC, mhl-28 possesses unique pharmacological properties that support its role in enhancing radiation response. Our experimental data show that mhl-28 markedly increases the formation of γ-H2AX foci and micronuclei—established markers of DNA damage—following irradiation. These findings suggest that mhl-28 actively interferes with the DNA damage response in the nucleus, thereby increasing the susceptibility of cancer cells to radiotherapy.

To elucidate the mechanisms underlying the synergistic effects of mhl-28 combined RT on cell proliferation, we investigated key mediators associated with the HDAC pathway, particularly those involved in cell cycle regulation. Radiation is known to inhibit cancer cell growth by inducing cell cycle arrest, predominantly at the G2/M phase—a critical checkpoint for DNA damage repair. Notably, a novel hybrid molecule, Roxyl-zhc-84, which dually targets HDAC and CDK, has been reported to induce G1 phase arrest in breast and ovarian cancer cell lines [47]. In our study, the combination of mhl-28 and RT significantly increased the proportion of cells arrested in the G2/M phase, indicating that mhl-28 enhances radiation-induced perturbations in cell cycle progression. These findings suggest that mhl-28 not only exacerbates radiation-induced DNA damage but also potentiates the cytotoxic efficacy of RT by prolonging cell retention in the DNA repair phase, thereby increasing susceptibility to radiation-mediated cell death.

The invasiveness of cancer cells is closely linked to their migratory potential. Localized RT can trigger the distant dissemination of residual tumor cells, thereby contributing to secondary tumor recurrence. Accumulating evidence indicates that hyperactivated STAT3 plays a pivotal role in driving this process. Furthermore, HDAC6 regulates ɑ-tubulin and its acetylation status, and its overexpression has been shown to enhance cell migration and motility [48]. Therefore, mhl-28 may modulate tumor cell motility by influencing STAT3 activity and the acetylation level of α-tubulin, potentially reducing the risk of tumor metastasis. Consistent with this hypothesis, the combination of mhl-28 and RT significantly suppressed the migration of A549 cells, highlighting its potential in preventing cancer metastasis and recurrence.

Our findings demonstrate that mhl-28 significantly exacerbates radiation-induced oxidative stress by disrupting redox homeostasis, marking a notable advancement over conventional HDAC inhibitors such as SAHA. While radiotherapy inherently generates ROS as a secondary mechanism of cellular damage [49], mhl-28 markedly amplifies this effect. Importantly, the observed increase in lipid ROS suggests that mhl-28 may selectively sensitize tumor cells to ferroptosis—a form of non-apoptotic cell death driven by iron-dependent lipid peroxidation. Although radiation alone induces only modest levels of lipid peroxidation, mhl-28 creates a conducive microenvironment that promotes the amplification of ferroptotic signaling.

It is crucial to investigate the modes of tumor cell death induced by the combination of mhl-28 and IR. Notably, apoptosis plays a significant role in this process. Through Hoechst 33,342 staining and Annexin V/PI double staining assays, we found that mhl-28 induces apoptosis in tumor cells in a dose-dependent manner. The distinct characteristics of various cell death pathways provide new opportunities for improving the efficacy of cancer therapies. Future studies should focus on elucidating the underlying molecular mechanisms, which may offer valuable clinical insights for cancer treatment. In conclusion, mhl-28 enhances tumor cell radiosensitivity by increasing oxidative stress and promoting apoptosis.

Importantly, the favorable safety profile of mhl-28 was further substantiated by comprehensive serum biochemistry. Quantitative analysis revealed no significant changes in markers of liver (ALT, AST) or kidney (CREA, BUN) function following treatment with mhl-28 alone or in combination with radiotherapy, compared to control animals. This lack of hepatorenal toxicity, combined with the observed stability in body weight and the absence of histopathological damage in vital organs, provides a multi-faceted and robust evidence base for the biosafety of mhl-28 at the therapeutic dose tested.

While our data robustly demonstrate that the combination of mhl-28 and RT yields a significantly enhanced antitumor effect in vitro and in vivo compared to either monotherapy, a formal quantitative analysis to precisely distinguish between synergistic and additive interactions—such as the Chou-Talalay Combination Index (CI) method—was beyond the primary scope of this initial efficacy and mechanism-exploration study. It is important to emphasize that the observed therapeutic enhancement, whether ultimately defined as additive or synergistic, holds substantial clinical promise, as it directly addresses the challenge of radioresistance. Future studies incorporating detailed dose-response matrices for both mhl-28 and radiation across multiple models will be essential to rigorously define the exact nature of this interaction and to optimize combination dosing schedules for translational development.

It is essential to acknowledge some limitations of this study. Firstly, the experiments were primarily conducted using X-rays, and the potential synergistic effects of other radiation modalities, such as g-rays and heavy ions, with mhl-28 remain largely unexplored. Second, further investigation is needed to elucidate the underlying mechanisms, particularly the specific role of mhl-28 in modulating the STAT3 and HDAC signaling pathways. Finally, clinical radiotherapy often involves diverse dose fractionation regimens, which significantly influence the induction of various forms of tumor cell death. The in vivo experiments in this study employed a single 8 Gy radiation dose, primarily aimed at efficiently evaluating the fundamental radiosensitizing effect of mhl-28. We acknowledge that standard clinical external beam radiotherapy typically involves fractionated schedules. Future studies will explore the efficacy and safety of mhl-28 in combination with more clinically relevant fractionated regimens (e.g., 2 Gy × multiple fractions). Furthermore, the high-dose-per-fraction model used here provides preliminary rationale for evaluating the potential of mhl-28 in enhancing emerging radiotherapy techniques such as stereotactic radiotherapy (SBRT/SRS).

## Conclusion

**Fig. 10:**
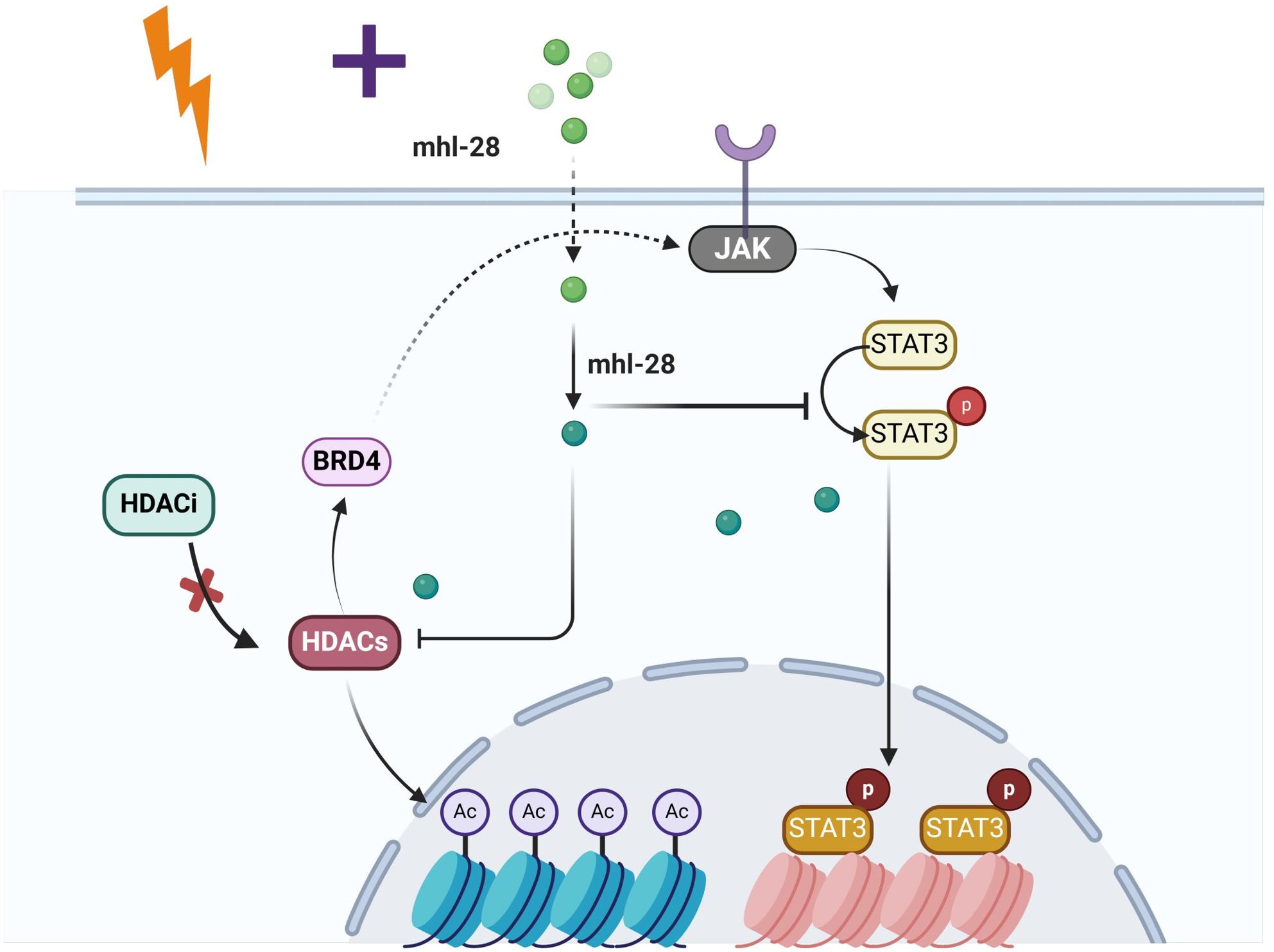
Model illustrating that mhl-28 overcomes Jak-STAT3-mediated drug resistance, addressing the limited response of traditional HDACi in solid tumors. Specifically, mhl-28 enhances the radiation sensitivity of tumor cells by inhibiting both STAT3 and HDAC, which are critical for understanding its full therapeutic potential

Our data indicate that mhl-28, a novel dual-targeting STAT3-HDAC inhibitor, exerts significant synergistic effects in suppressing tumor cell proliferation and enhancing radiosensitization (Fig. 10). By simultaneously modulating both STAT3 and HDAC pathways, mhl-28 overcomes the limitations associated with single-target inhibitors and potentiates the efficacy of radiotherapy. These findings support the rationale for combining HDAC and STAT3 inhibition with radiation therapy, paving the way toward an optimized therapeutic paradigm for the treatment of solid tumors and providing new insights into precision radiosensitization strategies.

## Materials and methods

### Cell lines, cell culture, and reagents

The A549, B16, HepG2, MCF-7, HeLa, HCT116, U87-MG, and KATO-III cell lines used in this study were obtained from the Cell Bank of the Chinese Academy of Sciences (Shanghai, China) and cultured under conditions of 5% CO_2_, 37 °C, and 95% humidity. All cell lines were maintained in DMEM medium (Gibco, USA, #C11995500BT) supplemented with 10% fetal bovine serum (Royacel, China, #RY-F22-01) and 1% penicillin-streptomycin (Gibco, USA, #15140122). Routine mycoplasma contamination testing was performed using a Mycoplasma Detection Kit (Yeasen, China, #40612ES60). The compounds SAHA and mhl-28 were stored at -40 °C in DMSO.

### Western blot analysis

Cells were lysed in RIPA buffer (Beyotime) containing protease inhibitors to obtain protein samples after drug treatment and/or irradiation. The samples were centrifuged at 4 °C for 10 min. Protein concentrations were determined using the BCA Protein Assay Kit (Yeasen, China, #20201ES90). Proteins were separated using 10% to 15% tris-tricine polyacrylamide gels (Yeasen, China, #20325, 20327) and then transferred to PVDF membranes (Bio-Rad, USA, #1620177). After blocking with 5% nonfat milk, membranes were incubated overnight at 4 °C with primary antibodies. Membranes were washed with TBST and incubated for an hour with horseradish peroxidase-conjugated goat anti-rabbit secondary antibodies (Proteintech) or goat anti-mouse secondary antibodies (ZSGB-BIO). Protein expression was measured using an enhanced chemiluminescence kit (Advansta). The following primary antibodies were used: H3, H4, HDAC1, HDAC6, STAT3, p-STAT3 (Tyr705), Ku70, Rad51, γ-H2AX, Bax, Bcl-2, N-cadherin, E-cadherin and GAPDH (Proteintech, China); DNA-PKcs, Ac-H3, Ac-H4, Ac-tubulin and Ac-α-tubulin (Cell Signaling Technology, USA).

### CCK-8 assay

Cell proliferation was assessed using the Cell Counting Kit-8 (CCK-8) assay (Yeasen). Approximately 5,000 cells were seeded per condition in 96-well plates (Corning, USA, #08620066). Cells were treated with 0.5 µM SAHA, mhl-28, or 0.1% DMSO in culture medium for 24, 48, or 72 h. After removing the drug-containing medium, fresh medium and 10 µL of CCK-8 reagent were added. After a 2-hour incubation, absorbance was measured at 450 nm using a microplate reader (Nano-800). Five parallel replicates were set up for each treatment group, and each experiment was performed in triplicate.

### Colony formation assay

Cells pre-treated with 0.5 µM SAHA, mhl-28, or 0.1% DMSO were seeded in 60 mm culture dishes at densities of 100 (0 Gy), 400 (2 Gy), 1000 (4 Gy), 2000 (6 Gy), and 4000 (8 Gy). After 14 days of incubation at 37 °C in DMEM medium, the cells were washed twice with PBS, fixed with 4% paraformaldehyde, and stained with 0.1% crystal violet for 20 min. Survival scores were calculated using a multi-target model, and survival curves were plotted using GraphPad Prism 9.5 (RRID: SCR_000306). The colony efficiency was calculated as follows: Colony Efficiency (%) = (Number of Colonies / Number of Seeded Cells) × 100%.

### Annexin V-FITC/PI dual staining for apoptosis analysis

Cells were treated with 0.5 µM mhl-28 or 0.1% DMSO for 24 h, followed by irradiation. After 24 h, the cells were digested with 0.25% trypsin (without EDTA) and washed twice with PBS. The cells were then collected and suspended in 500 µL of binding buffer. To this mixture, 5 µL of Annexin V-FITC and 10 µL of PI (Beyotime) were added, and the samples were incubated for 15 min in the dark. Apoptosis was analyzed using a BD FACSCalibur flow cytometer (BD Biosciences), and the data were processed with FlowJo software (FlowJo 10.5, LLC).

### Micronucleus assay

Cells were treated with 0.5 µM mhl-28 or 0.1% DMSO for 24 h, followed by irradiation. Approximately 60,000 to 80,000 cells per dish were seeded in 35 mm dishes and cultured at 37 °C for 24 h. The cells were fixed in Carnoy’s fixative (methanol: acetic acid = 3:1) for 10 min, washed with PBS three times, and allowed to air dry. A few drops of 0.01% acridine orange solution were added to the cell layer, covered with a clean coverslip, and the micronuclei were counted using a fluorescence microscope. The micronucleus rate was calculated as follows: Micronucleus Rate = (Number of Micronuclei / Total Cell Count) × 100%.

### Cell cycle analysis

Cells were treated with 0.5 µM mhl-28 or 0.1% DMSO for 24 h, then digested, collected, and fixed in 75% ice-cold ethanol at 4 °C for 24 h. After fixation, the cells were resuspended in PBS containing 50 µg/mL PI and 50 µg/mL RNase A. PI-stained cells were analyzed using a BD FACSCalibur flow cytometer (BD Biosciences), and the results were processed using FlowJo software (FlowJo 10.5, LLC).

### Hoechst staining for apoptosis detection

Approximately 60,000 to 80,000 cells per dish were seeded in 35 mm dishes and cultured at 37 °C for 24 h. Cells were then treated with varying concentrations of mhl-28 or 0.1% DMSO for an additional 24 h, followed by irradiation. After 24 h, the cells were washed with PBS, fixed with 0.5 mL of fixative solution, and kept at 4 °C overnight. The fixative solution was removed, and the cells were washed twice with PBS before adding 0.5 mL of Hoechst 33,342 staining solution (Beyotime). The cells were stained in the dark for 5 min and rinsed with PBS. A drop of anti-fluorescence quencher was added to the cell layer and covered with a clean coverslip. Apoptosis was observed and counted using a fluorescence microscope with an excitation wavelength of 350 nm and an emission wavelength of 460 nm.

### Scratch wound healing assay

Tumor cells were seeded at a density of 3 × 10^5^ per well in 12-well plates (Corning, USA, #12322601). After cells adhered, they were treated with varying concentrations of mhl-28 or 0.1% DMSO for 24 h. Following irradiation, a 200 µL pipette tip was used to carefully create a scratch across the cell monolayer perpendicular to the marked area. The plate was then washed three times with PBS and cultured further in serum-free medium. At 0, 12, 24, and 48 h post-injury, images of the scratch edges were captured using an electron microscope to analyze cell migration. The scratch area was quantified with Image J. The migration rate was calculated as follows: Migration Rate = (Scratch Area at 0 h - Scratch Area at 24–48 h) / (Scratch Area at 0 h) × 100%.

### Immunofluorescence for γ-H2AX Foci

Cells grown on glass coverslips were pretreated with 0.5 µM compounds for 24 h, irradiated (4 Gy, X-Rad 320), and fixed 0.5 h post-IR with 4% paraformaldehyde at 25 °C for 15 min. After permeabilization with 0.5% Triton X-100 for 10 min, samples were blocked with 5% BSA for 1 h and incubated with γ-H2AX antibody (1:1000, Proteintech, #Cat No. 68888-1-1 g) overnight at 4 °C. Alexa Fluor 594-conjugated and FITC-conjugated secondary antibody (1:100; YEASEN, #34912ES60 and 33207ES60) was applied for 1 h, with nuclei counterstained using DAPI (YEASEN, #36308ES20). Coverslips were mounted with Antifade Mountant and visualized under a confocal microscope.

### Transwell migration assay

Migration assays were conducted using 8-µm pore Transwell inserts (Corning, #3422). Cells were serum-starved for 12 h after 24-h drug pretreatment (0.5 µM mhl-28/SAHA). Following trypsinization, 1.5 × 10⁴ cells in serum-free medium were seeded into upper chambers. Lower chambers contained DMEM with 10% FBS chemoattractant. After 4 Gy irradiation, cells migrated for 24 h at 37 °C. Non-migrated cells were removed with cotton swabs. Migrated cells on the lower membrane surface were fixed with methanol, stained with 0.1% crystal violet for 15 min, and counted in five random fields/well.

### Ros kinetics measurement

Intracellular ROS was detected using DCFH-DA probe (Beyotime, #S0033S). After 24-h drug pretreatment and 4 Gy irradiation, cells in 96-well plates were immediately loaded with 10 µM DCFH-DA in PBS for 30 min. Fluorescence intensity (Ex/Em = 488/525 nm) was recorded at 10-min intervals for 120 min using microplate reader. ROS generation rate was calculated as the slope of fluorescence intensity curve (0–120 min).

### Lipid peroxidation analysis via flow cytometry

Lipid ROS was assessed using C11-BODIPY^581/591^ (Invitrogen, Cat#D3861). Cells pretreated 24 h with compounds received 10 Gy irradiation. After 48 h incubation, cells were trypsinized and stained with 5 µM C11-BODIPY in serum-free medium for 30 min at 37 °C. Flow cytometry analyzed 10,000 events/sample using 488 nm excitation with emission captured at 530 nm (oxidized) and 590 nm (reduced) channels. Lipid peroxidation index was calculated as FITC/PE fluorescence ratio (FITC-A: FL1 channel; PE-A: FL2 channel) with analysis in FlowJo v10.6.

### Irradiation

For cell irradiation, an X-Rad 320 biological irradiator (Precision X-Ray, Greenville, South Carolina, USA) was used at a dose rate of 300 cGy/min. For mouse irradiation, mice were anesthetized before irradiation. To avoid unnecessary radiation exposure to other organs, only the hind leg with the implanted tumor was positioned in the radiation field (32 cm × 7 cm) by adjusting the collimator jaws.

### Subcutaneous xenograft mouse model

Four-week-old female C57BL/6 mice used in this study were purchased from Zhejiang Viton Lihua Laboratory Animal Technology Co., Ltd. The care and management of laboratory animals were in accordance with the Guide for the Care and Use of Laboratory Animals. All procedures complied with the standards of the Zhejiang Provincial Animal Committee and the guidelines of the Wenzhou Medical University Ethics Committee (Number: wydw2023-0473). Mice were housed in a pathogen-free environment under a 12-hour light/dark cycle with humidity levels of 40–60% and temperatures of 22–24 °C, with access to sterile food and tap water.

For the mouse melanoma model, 1 × 10^6^ B16 cells were subcutaneously injected into the right hind leg of C57BL/6 mice. On day 5 post-injection, mice were randomized into groups of six. On days 6, 7, and 8 post-injection, mice received intraperitoneal injections of either mhl-28 (15 mg/kg), SAHA (15 mg/kg), or PBS. Both mhl-28 and SAHA were dissolved in a mixture of 10% β-cyclodextrin, 10% acetylated castor oil, and 80% saline to form a 3 mg/mL solution. On day 7, the irradiated groups were exposed to 8 Gy X-rays. Tumor size was measured with calipers every two days, and tumor volume was calculated using the formula: V = 0.5 × a² × b (where “a” is tumor length and “b” is tumor width). After 21 days, all mice were sacrificed, and tumors were isolated, weighed, and stored at -80 °C for further analysis.

### Histopathological analysis

Following 21 days of treatment, all mice were humanely euthanized via cervical dislocation under isoflurane anesthesia. Primary tumors and vital organs (heart, liver) were immediately harvested and fixed in 10% neutral-buffered formalin for 48 h at 4 °C. Tissues were processed through graded ethanol series, embedded in paraffin (Leica, Germany, #39601006), and sectioned at 4 μm thickness using a rotary microtome (Leica RM2235). For hematoxylin and eosin (H&E) staining, sections were stained with Mayer’s hematoxylin for 5 min and eosin Y for 90 s (Beyotime, China, #C0105S). Immunohistochemistry using primary antibodies against Ki-67 (1:1000, Proteintech, #Cat No. 27309-1-AP) and γ-H2AX (1:1000, Proteintech, #Cat No. 68888-1-1 g) with DAB visualization (Beyotime, China, #P0203).

### Serum biochemical analysis

At the experimental endpoint (day 21), blood was collected from mice via retro-orbital bleeding under anesthesia. The blood samples were allowed to clot at room temperature for 1 h and then centrifuged at 4 °C, 3000 rpm for 15 min to obtain serum. Serum levels of alanine aminotransferase (ALT), aspartate aminotransferase (AST), creatinine (CREA), and blood urea nitrogen (BUN) were quantitatively measured using a fully automated biochemical analyzer (Beckman Coulter AU480]) with corresponding commercial assay kits, following the manufacturer’s protocols.

### Statistical analysis

All statistical analyses were conducted using SPSS version 19.0 and GraphPad Prism 5.0 software. Data are presented as mean ± standard deviation. For comparisons among multiple groups, One-Way ANOVA was used, followed by LSD t-tests for pairwise comparisons. A p-value of less than 0.05 or 0.01 was considered statistically significant.

## Supplementary Information

Supplementary Material 1: Extended Fig. 1：Densitometric quantification of Western blot analyses presented in Fig. 1A. Quantification of p-STAT3 (Tyr705) levels normalized to total STAT3 in A549 and B16 from Fig. 1A. Extended Fig. 2：Densitometric quantification of Western blot analyses presented in Fig. 1B. Quantification of Ac-Tub、Ac-α-Tub、Ac-H3/H3、Ac-H4/H4 levels in A549 and B16 from Fig. 1B. Extended Fig. 3：Densitometric quantification of Western blot analyses presented in Fig. 1C. Quantification of p-STAT3 (Tyr705) levels normalized to total STAT3 in A549 and B16 from Fig. 1C. Extended Fig. 4：Densitometric quantification of Western blot analyses presented in Fig. 1D. Quantification of Ac-Tub、Ac-α-Tub、Ac-H3/H3、Ac-H4/H4 levels in A549 and B16 from Fig. 1D. Extended Fig. 5: mhl-28 inhibits tumor cell proliferation. Cell viability in A549 (A), MDA-MB-231 (B) , and B16 (C) cells assessed using CCK8 assay after 48 hours of treatment with different concentration of mhl-28. The vehicle control group was treated with 0.1% DMSO. Data are presented as mean ± SD from three independent experiments (n=3). 'ns' indicates no statistical significance, * P < 0.05, ** P < 0.01, *** P < 0.001, and **** P < 0.0001. Extended Fig. 6: Representative images of colony formation (A) and quantified survival fractions (B) in A549 cells treated with various concentrations of mhl-28 and SAHA. Data are presented as mean ± SD from three independent experiments (n=3). Statistical significance: Asterisks above bars (*, **, ***, ****) indicate comparisons with the consentration-only control group at the same dose. Asterisks above brackets (*, **, ***, ****) indicate pairwise comparisons within the bracketed groups. 'ns' indicates no statistical significance. * P < 0.05, ** P < 0.01, *** P < 0.001, **** P < 0.0001. Extended Fig. 7: Densitometric quantification of Western blot analyses presented in Fig. 4G. Quantification of DNA-PKcs、Ku70、Rad51、γ-H2AX levels in A549 and B16 from Fig. 4G. Extended Fig. 8: (A) Representative images of cell migration at 0 h and 24 h after scratch assay in A549 cells treated with various concentrations of mhl-28 and SAHA for 48 hours; quantification of scratch healing rates (B). (C) Representative images of cell migration at 0 h, 24 h, and 48 h after scratch assay in A549 cells treated with 0.5 μM mhl-28 for 24 hours and exposed to 0, 4, or 8 Gy of X-rays; quantification of scratch healing rates (D). The vehicle control group was treated with 0.1% DMSO. Data are presented as mean ± SD from three independent experiments (n=3). Statistical significance: Asterisks above bars (*, **, ***, ****) indicate comparisons with the consentration/IR-only control group at the same dose. Asterisks above brackets (*, **, ***, ****) indicate pairwise comparisons within the bracketed groups. 'ns' indicates no statistical significance. * P < 0.05, ** P < 0.01, *** P < 0.001, **** P < 0.0001. Extended Fig. 9: Densitometric quantification of Western blot analyses presented in Fig. 6I. Quantification of Bax/Bcl-2 levels in A549 and B16 from Fig. 6I. Extended Fig. 10: Densitometric quantification of Western blot analyses presented in Fig. 7C. Quantification of ACSL4、SLC7A11、GPX4、Nrf2、TRX1 levels in A549 and B16 from Fig. 7C. Extended Fig. 11: mhl-28 does not induce hepatorenal toxicity in mice. Serum biochemical analysis of (A) alanine aminotransferase (ALT), (B) aspartate aminotransferase (AST), (C) creatinine (CREA), and (D) blood urea nitrogen (BUN) in mice from different treatment groups at the study endpoint (day 21). Groups: PBS (vehicle control), mhl-28 (15 mg/kg), IR (8 Gy X-ray), and mhl-28 + IR (combination). Data are presented as mean ± SD (n = 6 mice per group). Statistical significance was determined by one-way ANOVA followed by LSD post-hoc test; ns indicates no significant difference (p > 0.05) between the indicated group and the PBS control group.

Supplementary Material 2.

## Data Availability

All data supporting the findings of this study are available within the paper and its Supplementary Information.

## Abbreviations

STAT3: Signal transducer and activator of transcription 3
HDAC: Histone deacetylase
IAL: Isoalantolactone
SAHA: Vorinostat
TCM: Traditional chinese medicine
HDACi: HDAC inhibitors
JAK: Janus kinase
BRD4: Bromodomain containing 4
LIFR: Leukemia inhibitory factor receptor
RT: Radiotherapy
Ac-: Acetylation-
DMSO: Dimethyl sulfoxide
DSB: Double-strand break
IR: Ionizing radiation
NHEJ: Non-homologous end joining
HR: Homologous recombination
PI: Propidium iodide
ROS: Reactive oxygen species
DCFH-DA: 2’,7’-dichlorodihydrofluorescein diacetate
CCK-8: Cell Counting Kit-8
