# Supplementary figures and images for: The radiosensitizing effects of a STAT3/HDAC dual-target inhibitor derived from isoalantolactone in solid tumor models
Source: BMC Cancer. 2026 Mar 6;26:493. doi: 10.1186/s12885-026-15816-7 (PMC13088848; doi:10.1186/s12885-026-15816-7)

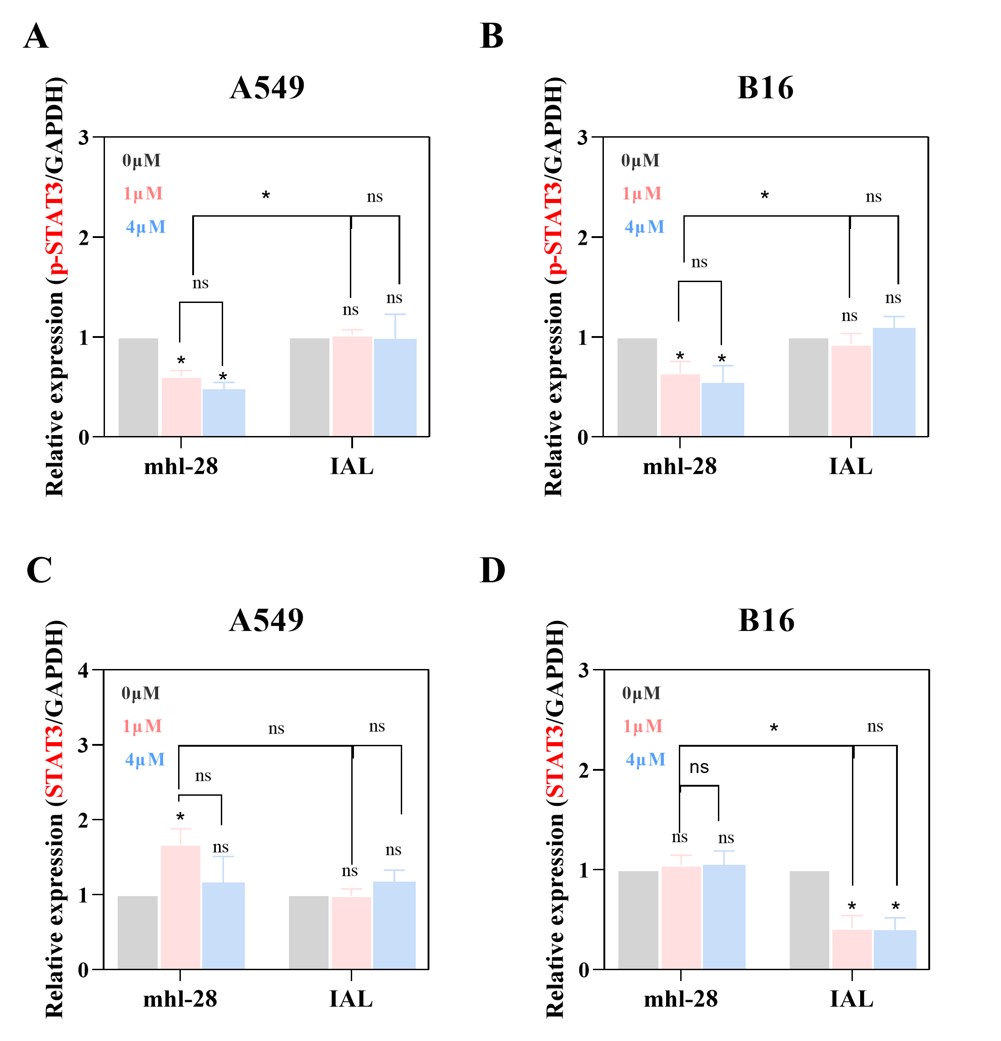

Supplement: Supplementary file 1 — Supplementary Material 1: Extended Fig. 1：Densitometric quantification of Western blot analyses presented in Fig. 1A. Quantification of p-STAT3 (Tyr705) levels normalized to total STAT3 in A549 and B16 from Fig. 1A. Extended Fig. 2：Densitometric quantification of Western blot analyses presented in Fig. 1B. Quantification of Ac-Tub、Ac-α-Tub、Ac-H3/H3、Ac-H4/H4 levels in A549 and B16 from Fig. 1B. Extended Fig. 3：Densitometric quantification of Western blot analyses presented in Fig. 1C. Quantification of p-STAT3 (Tyr705) levels normalized to total STAT3 in A549 and B16 from Fig. 1C. Extended Fig. 4：Densitometric quantification of Western blot analyses presented in Fig. 1D. Quantification of Ac-Tub、Ac-α-Tub、Ac-H3/H3、Ac-H4/H4 levels in A549 and B16 from Fig. 1D. Extended Fig. 5: mhl-28 inhibits tumor cell proliferation. Cell viability in A549 (A), MDA-MB-231 (B) , and B16 (C) cells assessed using CCK8 assay after 48 hours of treatment with different concentration of mhl-28. The vehicle control group was treated with 0.1% DMSO. Data are presented as mean ± SD from three independent experiments (n=3). 'ns' indicates no statistical significance, * P < 0.05, ** P < 0.01, *** P < 0.001, and **** P < 0.0001. Extended Fig. 6: Representative images of colony formation (A) and quantified survival fractions (B) in A549 cells treated with various concentrations of mhl-28 and SAHA. Data are presented as mean ± SD from three independent experiments (n=3). Statistical significance: Asterisks above bars (*, **, ***, ****) indicate comparisons with the consentration-only control group at the same dose. Asterisks above brackets (*, **, ***, ****) indicate pairwise comparisons within the bracketed groups. 'ns' indicates no statistical significance. * P < 0.05, ** P < 0.01, *** P < 0.001, **** P < 0.0001. Extended Fig. 7: Densitometric quantification of Western blot analyses presented in Fig. 4G. Quantification of DNA-PKcs、Ku70、Rad51、γ-H2AX levels in A549 and B16 from Fig. 4G. Extended Fig [file 12885_2026_15816_MOESM1_ESM.zip › Extend Fig 1.jpg]

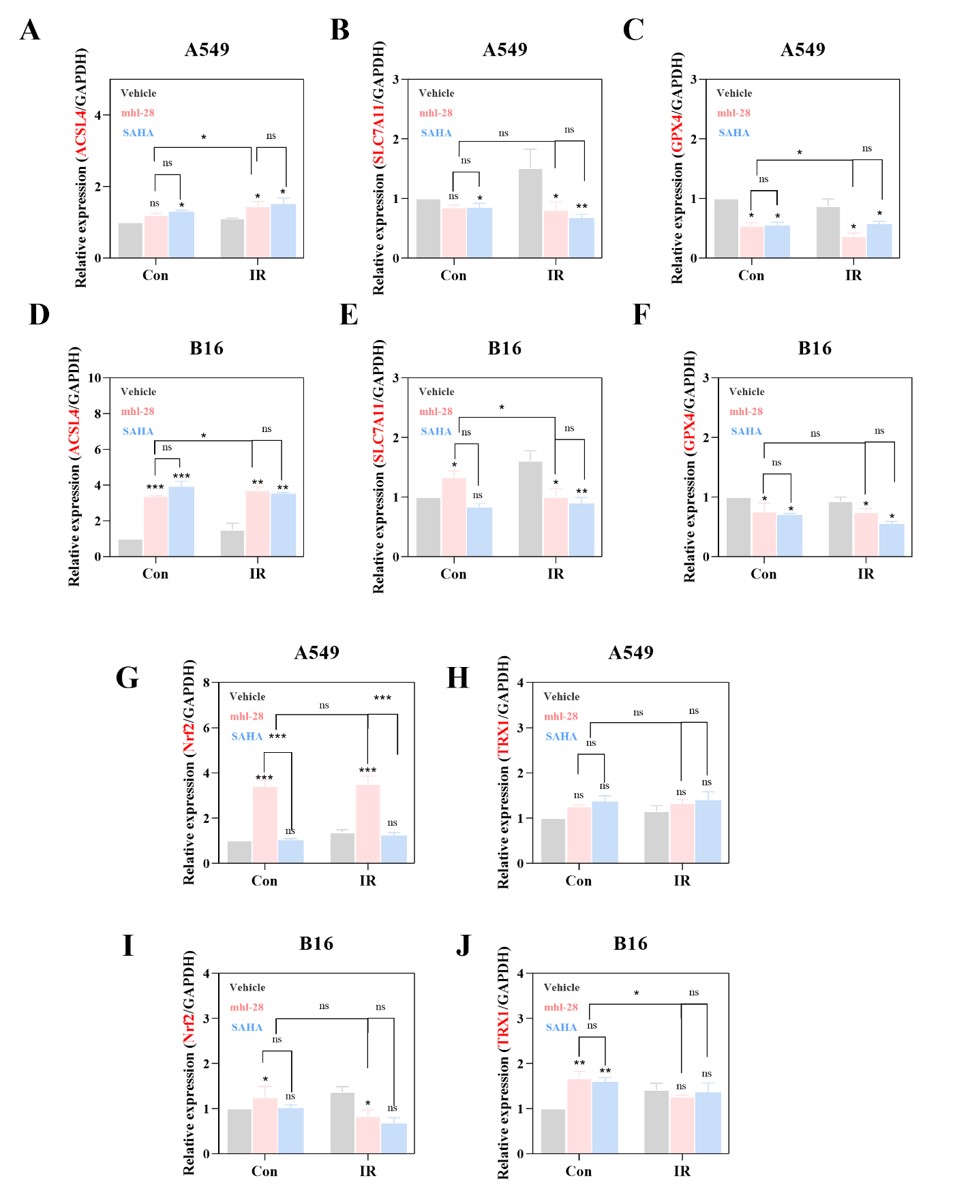

Supplement: Supplementary file 1 — Supplementary Material 1: Extended Fig. 1：Densitometric quantification of Western blot analyses presented in Fig. 1A. Quantification of p-STAT3 (Tyr705) levels normalized to total STAT3 in A549 and B16 from Fig. 1A. Extended Fig. 2：Densitometric quantification of Western blot analyses presented in Fig. 1B. Quantification of Ac-Tub、Ac-α-Tub、Ac-H3/H3、Ac-H4/H4 levels in A549 and B16 from Fig. 1B. Extended Fig. 3：Densitometric quantification of Western blot analyses presented in Fig. 1C. Quantification of p-STAT3 (Tyr705) levels normalized to total STAT3 in A549 and B16 from Fig. 1C. Extended Fig. 4：Densitometric quantification of Western blot analyses presented in Fig. 1D. Quantification of Ac-Tub、Ac-α-Tub、Ac-H3/H3、Ac-H4/H4 levels in A549 and B16 from Fig. 1D. Extended Fig. 5: mhl-28 inhibits tumor cell proliferation. Cell viability in A549 (A), MDA-MB-231 (B) , and B16 (C) cells assessed using CCK8 assay after 48 hours of treatment with different concentration of mhl-28. The vehicle control group was treated with 0.1% DMSO. Data are presented as mean ± SD from three independent experiments (n=3). 'ns' indicates no statistical significance, * P < 0.05, ** P < 0.01, *** P < 0.001, and **** P < 0.0001. Extended Fig. 6: Representative images of colony formation (A) and quantified survival fractions (B) in A549 cells treated with various concentrations of mhl-28 and SAHA. Data are presented as mean ± SD from three independent experiments (n=3). Statistical significance: Asterisks above bars (*, **, ***, ****) indicate comparisons with the consentration-only control group at the same dose. Asterisks above brackets (*, **, ***, ****) indicate pairwise comparisons within the bracketed groups. 'ns' indicates no statistical significance. * P < 0.05, ** P < 0.01, *** P < 0.001, **** P < 0.0001. Extended Fig. 7: Densitometric quantification of Western blot analyses presented in Fig. 4G. Quantification of DNA-PKcs、Ku70、Rad51、γ-H2AX levels in A549 and B16 from Fig. 4G. Extended Fig [file 12885_2026_15816_MOESM1_ESM.zip › Extend Fig 10.jpg]

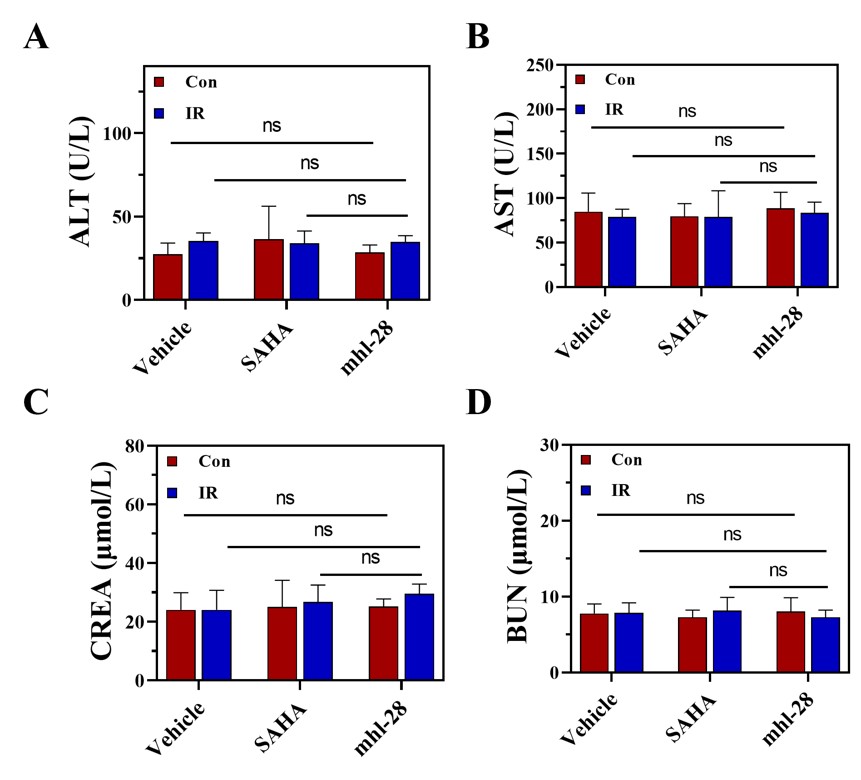

Supplement: Supplementary file 1 — Supplementary Material 1: Extended Fig. 1：Densitometric quantification of Western blot analyses presented in Fig. 1A. Quantification of p-STAT3 (Tyr705) levels normalized to total STAT3 in A549 and B16 from Fig. 1A. Extended Fig. 2：Densitometric quantification of Western blot analyses presented in Fig. 1B. Quantification of Ac-Tub、Ac-α-Tub、Ac-H3/H3、Ac-H4/H4 levels in A549 and B16 from Fig. 1B. Extended Fig. 3：Densitometric quantification of Western blot analyses presented in Fig. 1C. Quantification of p-STAT3 (Tyr705) levels normalized to total STAT3 in A549 and B16 from Fig. 1C. Extended Fig. 4：Densitometric quantification of Western blot analyses presented in Fig. 1D. Quantification of Ac-Tub、Ac-α-Tub、Ac-H3/H3、Ac-H4/H4 levels in A549 and B16 from Fig. 1D. Extended Fig. 5: mhl-28 inhibits tumor cell proliferation. Cell viability in A549 (A), MDA-MB-231 (B) , and B16 (C) cells assessed using CCK8 assay after 48 hours of treatment with different concentration of mhl-28. The vehicle control group was treated with 0.1% DMSO. Data are presented as mean ± SD from three independent experiments (n=3). 'ns' indicates no statistical significance, * P < 0.05, ** P < 0.01, *** P < 0.001, and **** P < 0.0001. Extended Fig. 6: Representative images of colony formation (A) and quantified survival fractions (B) in A549 cells treated with various concentrations of mhl-28 and SAHA. Data are presented as mean ± SD from three independent experiments (n=3). Statistical significance: Asterisks above bars (*, **, ***, ****) indicate comparisons with the consentration-only control group at the same dose. Asterisks above brackets (*, **, ***, ****) indicate pairwise comparisons within the bracketed groups. 'ns' indicates no statistical significance. * P < 0.05, ** P < 0.01, *** P < 0.001, **** P < 0.0001. Extended Fig. 7: Densitometric quantification of Western blot analyses presented in Fig. 4G. Quantification of DNA-PKcs、Ku70、Rad51、γ-H2AX levels in A549 and B16 from Fig. 4G. Extended Fig [file 12885_2026_15816_MOESM1_ESM.zip › Extend Fig 11.jpg]

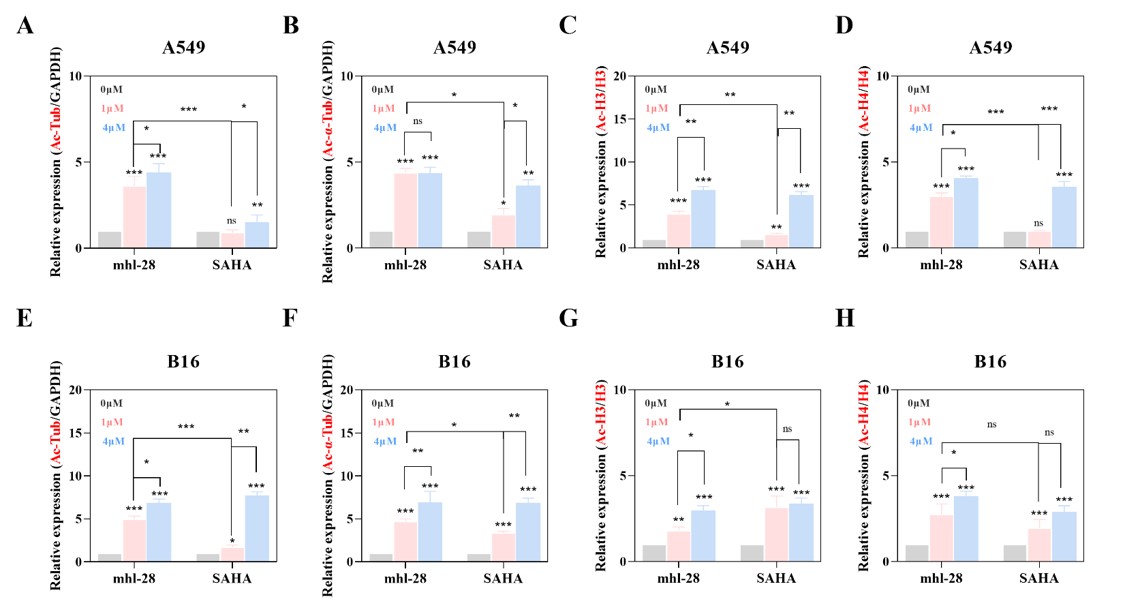

Supplement: Supplementary file 1 — Supplementary Material 1: Extended Fig. 1：Densitometric quantification of Western blot analyses presented in Fig. 1A. Quantification of p-STAT3 (Tyr705) levels normalized to total STAT3 in A549 and B16 from Fig. 1A. Extended Fig. 2：Densitometric quantification of Western blot analyses presented in Fig. 1B. Quantification of Ac-Tub、Ac-α-Tub、Ac-H3/H3、Ac-H4/H4 levels in A549 and B16 from Fig. 1B. Extended Fig. 3：Densitometric quantification of Western blot analyses presented in Fig. 1C. Quantification of p-STAT3 (Tyr705) levels normalized to total STAT3 in A549 and B16 from Fig. 1C. Extended Fig. 4：Densitometric quantification of Western blot analyses presented in Fig. 1D. Quantification of Ac-Tub、Ac-α-Tub、Ac-H3/H3、Ac-H4/H4 levels in A549 and B16 from Fig. 1D. Extended Fig. 5: mhl-28 inhibits tumor cell proliferation. Cell viability in A549 (A), MDA-MB-231 (B) , and B16 (C) cells assessed using CCK8 assay after 48 hours of treatment with different concentration of mhl-28. The vehicle control group was treated with 0.1% DMSO. Data are presented as mean ± SD from three independent experiments (n=3). 'ns' indicates no statistical significance, * P < 0.05, ** P < 0.01, *** P < 0.001, and **** P < 0.0001. Extended Fig. 6: Representative images of colony formation (A) and quantified survival fractions (B) in A549 cells treated with various concentrations of mhl-28 and SAHA. Data are presented as mean ± SD from three independent experiments (n=3). Statistical significance: Asterisks above bars (*, **, ***, ****) indicate comparisons with the consentration-only control group at the same dose. Asterisks above brackets (*, **, ***, ****) indicate pairwise comparisons within the bracketed groups. 'ns' indicates no statistical significance. * P < 0.05, ** P < 0.01, *** P < 0.001, **** P < 0.0001. Extended Fig. 7: Densitometric quantification of Western blot analyses presented in Fig. 4G. Quantification of DNA-PKcs、Ku70、Rad51、γ-H2AX levels in A549 and B16 from Fig. 4G. Extended Fig [file 12885_2026_15816_MOESM1_ESM.zip › Extend Fig 2.jpg]

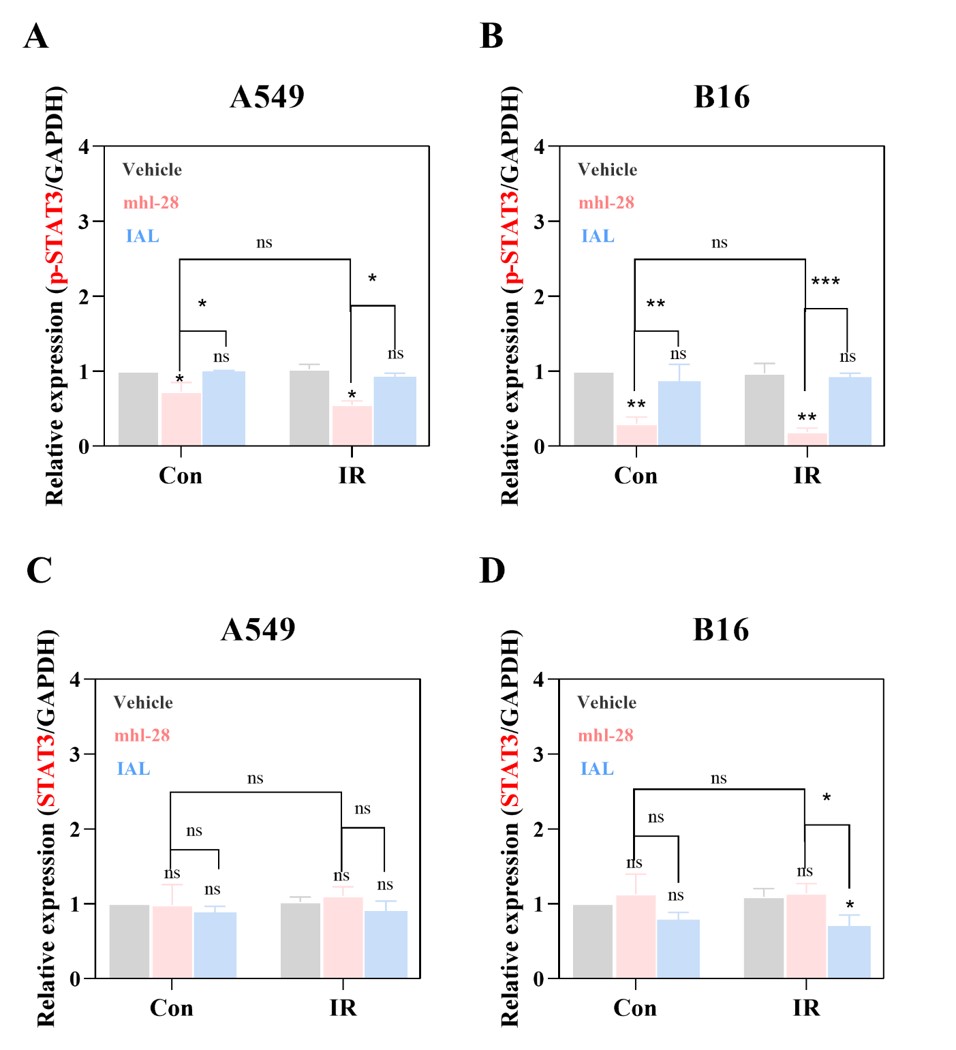

Supplement: Supplementary file 1 — Supplementary Material 1: Extended Fig. 1：Densitometric quantification of Western blot analyses presented in Fig. 1A. Quantification of p-STAT3 (Tyr705) levels normalized to total STAT3 in A549 and B16 from Fig. 1A. Extended Fig. 2：Densitometric quantification of Western blot analyses presented in Fig. 1B. Quantification of Ac-Tub、Ac-α-Tub、Ac-H3/H3、Ac-H4/H4 levels in A549 and B16 from Fig. 1B. Extended Fig. 3：Densitometric quantification of Western blot analyses presented in Fig. 1C. Quantification of p-STAT3 (Tyr705) levels normalized to total STAT3 in A549 and B16 from Fig. 1C. Extended Fig. 4：Densitometric quantification of Western blot analyses presented in Fig. 1D. Quantification of Ac-Tub、Ac-α-Tub、Ac-H3/H3、Ac-H4/H4 levels in A549 and B16 from Fig. 1D. Extended Fig. 5: mhl-28 inhibits tumor cell proliferation. Cell viability in A549 (A), MDA-MB-231 (B) , and B16 (C) cells assessed using CCK8 assay after 48 hours of treatment with different concentration of mhl-28. The vehicle control group was treated with 0.1% DMSO. Data are presented as mean ± SD from three independent experiments (n=3). 'ns' indicates no statistical significance, * P < 0.05, ** P < 0.01, *** P < 0.001, and **** P < 0.0001. Extended Fig. 6: Representative images of colony formation (A) and quantified survival fractions (B) in A549 cells treated with various concentrations of mhl-28 and SAHA. Data are presented as mean ± SD from three independent experiments (n=3). Statistical significance: Asterisks above bars (*, **, ***, ****) indicate comparisons with the consentration-only control group at the same dose. Asterisks above brackets (*, **, ***, ****) indicate pairwise comparisons within the bracketed groups. 'ns' indicates no statistical significance. * P < 0.05, ** P < 0.01, *** P < 0.001, **** P < 0.0001. Extended Fig. 7: Densitometric quantification of Western blot analyses presented in Fig. 4G. Quantification of DNA-PKcs、Ku70、Rad51、γ-H2AX levels in A549 and B16 from Fig. 4G. Extended Fig [file 12885_2026_15816_MOESM1_ESM.zip › Extend Fig 3.jpg]

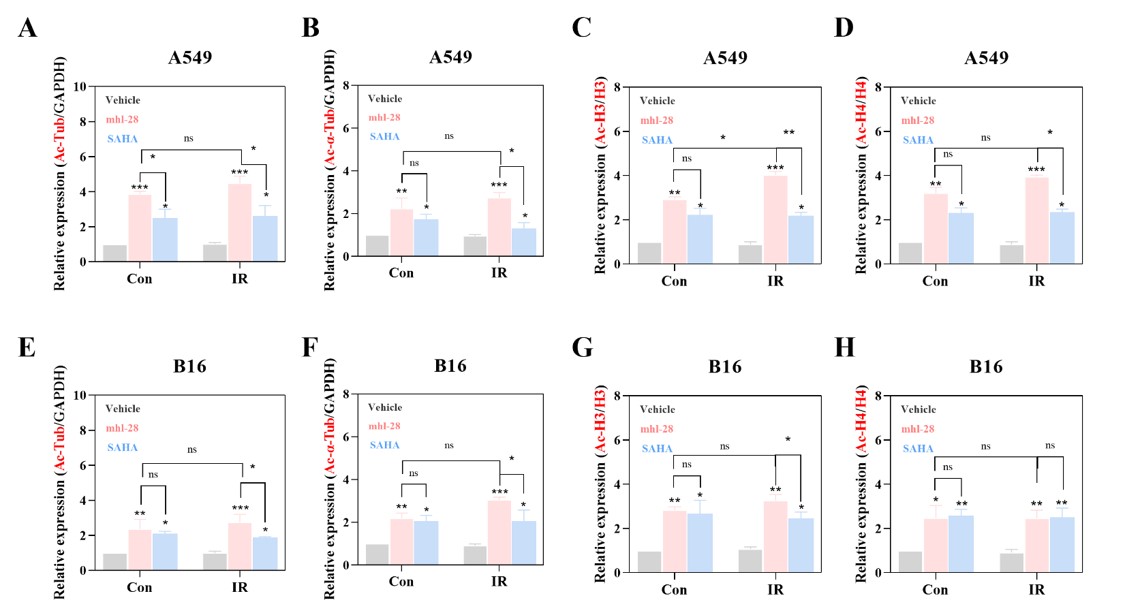

Supplement: Supplementary file 1 — Supplementary Material 1: Extended Fig. 1：Densitometric quantification of Western blot analyses presented in Fig. 1A. Quantification of p-STAT3 (Tyr705) levels normalized to total STAT3 in A549 and B16 from Fig. 1A. Extended Fig. 2：Densitometric quantification of Western blot analyses presented in Fig. 1B. Quantification of Ac-Tub、Ac-α-Tub、Ac-H3/H3、Ac-H4/H4 levels in A549 and B16 from Fig. 1B. Extended Fig. 3：Densitometric quantification of Western blot analyses presented in Fig. 1C. Quantification of p-STAT3 (Tyr705) levels normalized to total STAT3 in A549 and B16 from Fig. 1C. Extended Fig. 4：Densitometric quantification of Western blot analyses presented in Fig. 1D. Quantification of Ac-Tub、Ac-α-Tub、Ac-H3/H3、Ac-H4/H4 levels in A549 and B16 from Fig. 1D. Extended Fig. 5: mhl-28 inhibits tumor cell proliferation. Cell viability in A549 (A), MDA-MB-231 (B) , and B16 (C) cells assessed using CCK8 assay after 48 hours of treatment with different concentration of mhl-28. The vehicle control group was treated with 0.1% DMSO. Data are presented as mean ± SD from three independent experiments (n=3). 'ns' indicates no statistical significance, * P < 0.05, ** P < 0.01, *** P < 0.001, and **** P < 0.0001. Extended Fig. 6: Representative images of colony formation (A) and quantified survival fractions (B) in A549 cells treated with various concentrations of mhl-28 and SAHA. Data are presented as mean ± SD from three independent experiments (n=3). Statistical significance: Asterisks above bars (*, **, ***, ****) indicate comparisons with the consentration-only control group at the same dose. Asterisks above brackets (*, **, ***, ****) indicate pairwise comparisons within the bracketed groups. 'ns' indicates no statistical significance. * P < 0.05, ** P < 0.01, *** P < 0.001, **** P < 0.0001. Extended Fig. 7: Densitometric quantification of Western blot analyses presented in Fig. 4G. Quantification of DNA-PKcs、Ku70、Rad51、γ-H2AX levels in A549 and B16 from Fig. 4G. Extended Fig [file 12885_2026_15816_MOESM1_ESM.zip › Extend Fig 4.jpg]

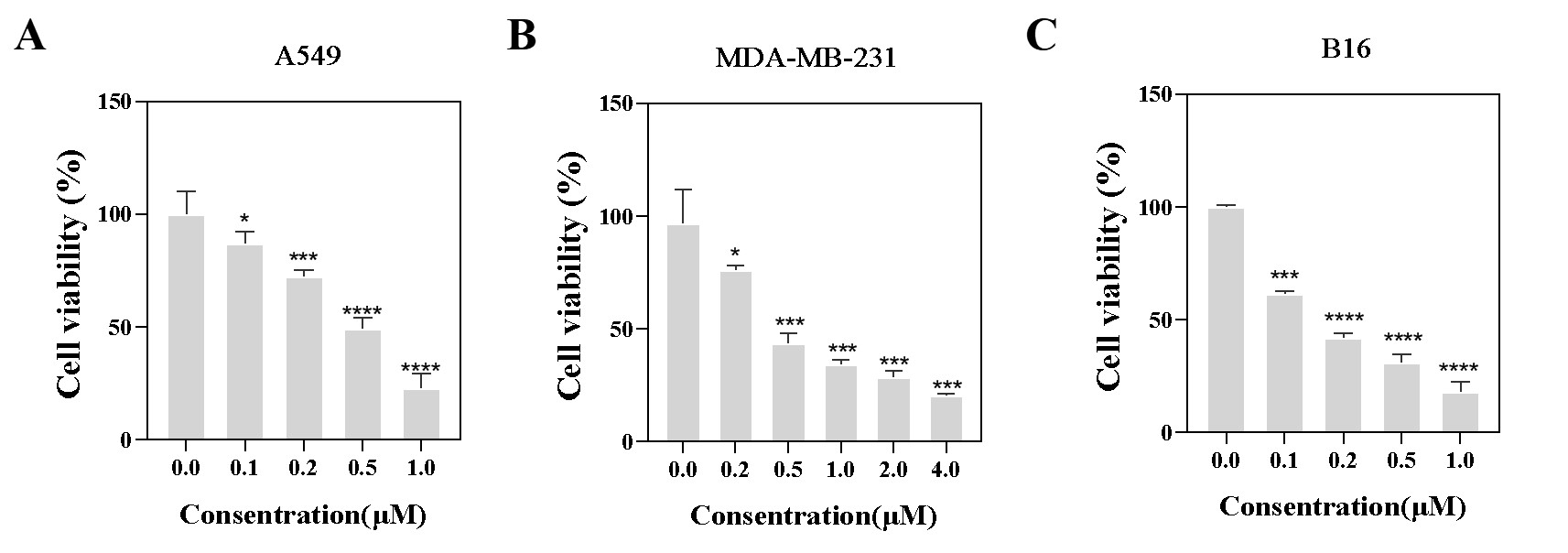

Supplement: Supplementary file 1 — Supplementary Material 1: Extended Fig. 1：Densitometric quantification of Western blot analyses presented in Fig. 1A. Quantification of p-STAT3 (Tyr705) levels normalized to total STAT3 in A549 and B16 from Fig. 1A. Extended Fig. 2：Densitometric quantification of Western blot analyses presented in Fig. 1B. Quantification of Ac-Tub、Ac-α-Tub、Ac-H3/H3、Ac-H4/H4 levels in A549 and B16 from Fig. 1B. Extended Fig. 3：Densitometric quantification of Western blot analyses presented in Fig. 1C. Quantification of p-STAT3 (Tyr705) levels normalized to total STAT3 in A549 and B16 from Fig. 1C. Extended Fig. 4：Densitometric quantification of Western blot analyses presented in Fig. 1D. Quantification of Ac-Tub、Ac-α-Tub、Ac-H3/H3、Ac-H4/H4 levels in A549 and B16 from Fig. 1D. Extended Fig. 5: mhl-28 inhibits tumor cell proliferation. Cell viability in A549 (A), MDA-MB-231 (B) , and B16 (C) cells assessed using CCK8 assay after 48 hours of treatment with different concentration of mhl-28. The vehicle control group was treated with 0.1% DMSO. Data are presented as mean ± SD from three independent experiments (n=3). 'ns' indicates no statistical significance, * P < 0.05, ** P < 0.01, *** P < 0.001, and **** P < 0.0001. Extended Fig. 6: Representative images of colony formation (A) and quantified survival fractions (B) in A549 cells treated with various concentrations of mhl-28 and SAHA. Data are presented as mean ± SD from three independent experiments (n=3). Statistical significance: Asterisks above bars (*, **, ***, ****) indicate comparisons with the consentration-only control group at the same dose. Asterisks above brackets (*, **, ***, ****) indicate pairwise comparisons within the bracketed groups. 'ns' indicates no statistical significance. * P < 0.05, ** P < 0.01, *** P < 0.001, **** P < 0.0001. Extended Fig. 7: Densitometric quantification of Western blot analyses presented in Fig. 4G. Quantification of DNA-PKcs、Ku70、Rad51、γ-H2AX levels in A549 and B16 from Fig. 4G. Extended Fig [file 12885_2026_15816_MOESM1_ESM.zip › Extend Fig 5.jpg]

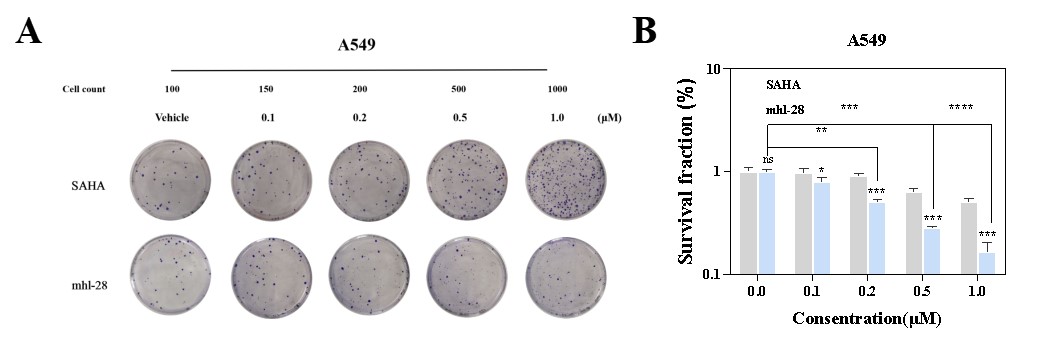

Supplement: Supplementary file 1 — Supplementary Material 1: Extended Fig. 1：Densitometric quantification of Western blot analyses presented in Fig. 1A. Quantification of p-STAT3 (Tyr705) levels normalized to total STAT3 in A549 and B16 from Fig. 1A. Extended Fig. 2：Densitometric quantification of Western blot analyses presented in Fig. 1B. Quantification of Ac-Tub、Ac-α-Tub、Ac-H3/H3、Ac-H4/H4 levels in A549 and B16 from Fig. 1B. Extended Fig. 3：Densitometric quantification of Western blot analyses presented in Fig. 1C. Quantification of p-STAT3 (Tyr705) levels normalized to total STAT3 in A549 and B16 from Fig. 1C. Extended Fig. 4：Densitometric quantification of Western blot analyses presented in Fig. 1D. Quantification of Ac-Tub、Ac-α-Tub、Ac-H3/H3、Ac-H4/H4 levels in A549 and B16 from Fig. 1D. Extended Fig. 5: mhl-28 inhibits tumor cell proliferation. Cell viability in A549 (A), MDA-MB-231 (B) , and B16 (C) cells assessed using CCK8 assay after 48 hours of treatment with different concentration of mhl-28. The vehicle control group was treated with 0.1% DMSO. Data are presented as mean ± SD from three independent experiments (n=3). 'ns' indicates no statistical significance, * P < 0.05, ** P < 0.01, *** P < 0.001, and **** P < 0.0001. Extended Fig. 6: Representative images of colony formation (A) and quantified survival fractions (B) in A549 cells treated with various concentrations of mhl-28 and SAHA. Data are presented as mean ± SD from three independent experiments (n=3). Statistical significance: Asterisks above bars (*, **, ***, ****) indicate comparisons with the consentration-only control group at the same dose. Asterisks above brackets (*, **, ***, ****) indicate pairwise comparisons within the bracketed groups. 'ns' indicates no statistical significance. * P < 0.05, ** P < 0.01, *** P < 0.001, **** P < 0.0001. Extended Fig. 7: Densitometric quantification of Western blot analyses presented in Fig. 4G. Quantification of DNA-PKcs、Ku70、Rad51、γ-H2AX levels in A549 and B16 from Fig. 4G. Extended Fig [file 12885_2026_15816_MOESM1_ESM.zip › Extend Fig 6.jpg]

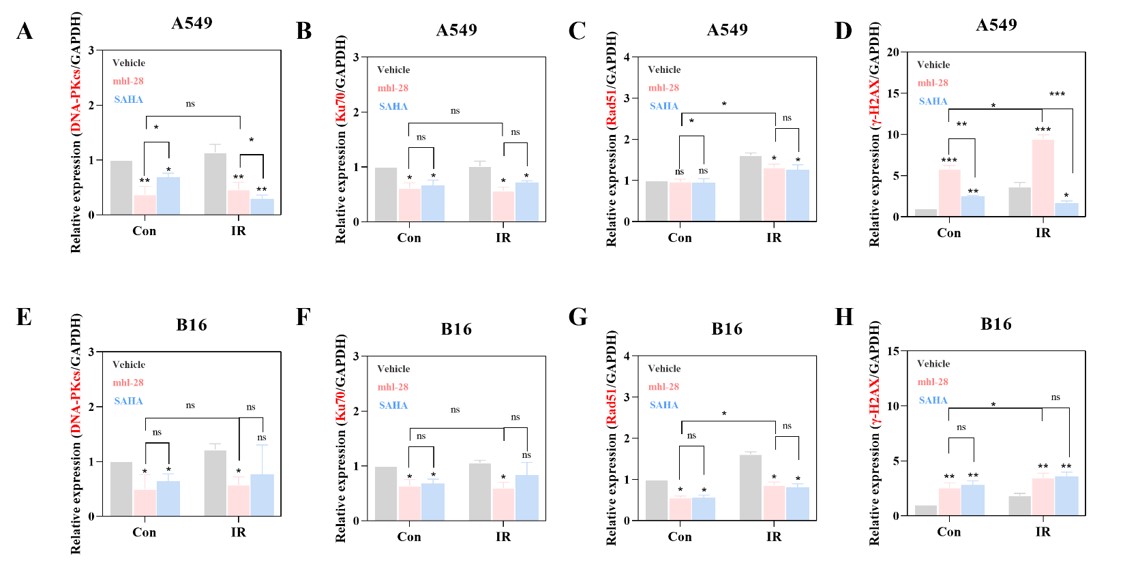

Supplement: Supplementary file 1 — Supplementary Material 1: Extended Fig. 1：Densitometric quantification of Western blot analyses presented in Fig. 1A. Quantification of p-STAT3 (Tyr705) levels normalized to total STAT3 in A549 and B16 from Fig. 1A. Extended Fig. 2：Densitometric quantification of Western blot analyses presented in Fig. 1B. Quantification of Ac-Tub、Ac-α-Tub、Ac-H3/H3、Ac-H4/H4 levels in A549 and B16 from Fig. 1B. Extended Fig. 3：Densitometric quantification of Western blot analyses presented in Fig. 1C. Quantification of p-STAT3 (Tyr705) levels normalized to total STAT3 in A549 and B16 from Fig. 1C. Extended Fig. 4：Densitometric quantification of Western blot analyses presented in Fig. 1D. Quantification of Ac-Tub、Ac-α-Tub、Ac-H3/H3、Ac-H4/H4 levels in A549 and B16 from Fig. 1D. Extended Fig. 5: mhl-28 inhibits tumor cell proliferation. Cell viability in A549 (A), MDA-MB-231 (B) , and B16 (C) cells assessed using CCK8 assay after 48 hours of treatment with different concentration of mhl-28. The vehicle control group was treated with 0.1% DMSO. Data are presented as mean ± SD from three independent experiments (n=3). 'ns' indicates no statistical significance, * P < 0.05, ** P < 0.01, *** P < 0.001, and **** P < 0.0001. Extended Fig. 6: Representative images of colony formation (A) and quantified survival fractions (B) in A549 cells treated with various concentrations of mhl-28 and SAHA. Data are presented as mean ± SD from three independent experiments (n=3). Statistical significance: Asterisks above bars (*, **, ***, ****) indicate comparisons with the consentration-only control group at the same dose. Asterisks above brackets (*, **, ***, ****) indicate pairwise comparisons within the bracketed groups. 'ns' indicates no statistical significance. * P < 0.05, ** P < 0.01, *** P < 0.001, **** P < 0.0001. Extended Fig. 7: Densitometric quantification of Western blot analyses presented in Fig. 4G. Quantification of DNA-PKcs、Ku70、Rad51、γ-H2AX levels in A549 and B16 from Fig. 4G. Extended Fig [file 12885_2026_15816_MOESM1_ESM.zip › Extend Fig 7.jpg]

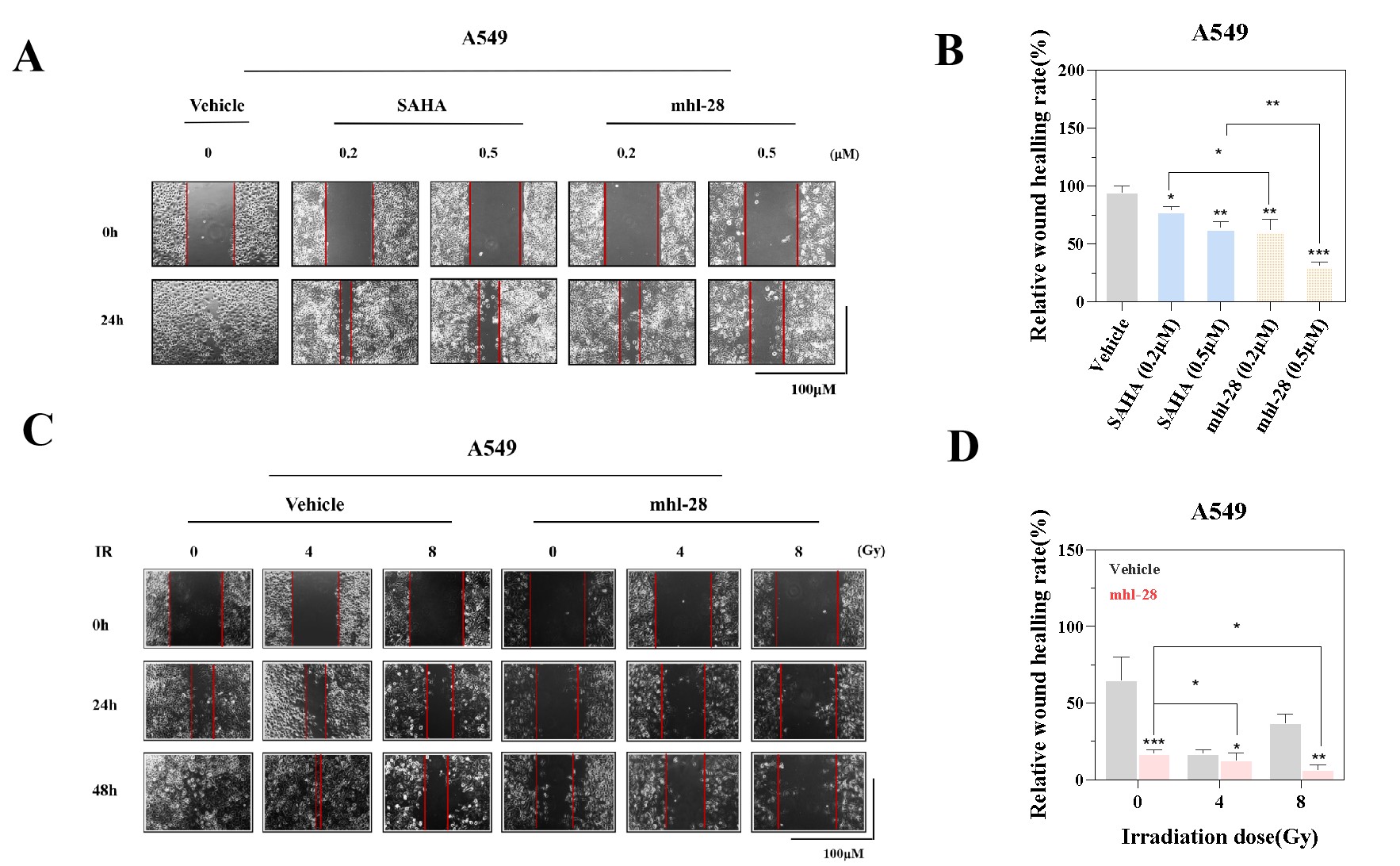

Supplement: Supplementary file 1 — Supplementary Material 1: Extended Fig. 1：Densitometric quantification of Western blot analyses presented in Fig. 1A. Quantification of p-STAT3 (Tyr705) levels normalized to total STAT3 in A549 and B16 from Fig. 1A. Extended Fig. 2：Densitometric quantification of Western blot analyses presented in Fig. 1B. Quantification of Ac-Tub、Ac-α-Tub、Ac-H3/H3、Ac-H4/H4 levels in A549 and B16 from Fig. 1B. Extended Fig. 3：Densitometric quantification of Western blot analyses presented in Fig. 1C. Quantification of p-STAT3 (Tyr705) levels normalized to total STAT3 in A549 and B16 from Fig. 1C. Extended Fig. 4：Densitometric quantification of Western blot analyses presented in Fig. 1D. Quantification of Ac-Tub、Ac-α-Tub、Ac-H3/H3、Ac-H4/H4 levels in A549 and B16 from Fig. 1D. Extended Fig. 5: mhl-28 inhibits tumor cell proliferation. Cell viability in A549 (A), MDA-MB-231 (B) , and B16 (C) cells assessed using CCK8 assay after 48 hours of treatment with different concentration of mhl-28. The vehicle control group was treated with 0.1% DMSO. Data are presented as mean ± SD from three independent experiments (n=3). 'ns' indicates no statistical significance, * P < 0.05, ** P < 0.01, *** P < 0.001, and **** P < 0.0001. Extended Fig. 6: Representative images of colony formation (A) and quantified survival fractions (B) in A549 cells treated with various concentrations of mhl-28 and SAHA. Data are presented as mean ± SD from three independent experiments (n=3). Statistical significance: Asterisks above bars (*, **, ***, ****) indicate comparisons with the consentration-only control group at the same dose. Asterisks above brackets (*, **, ***, ****) indicate pairwise comparisons within the bracketed groups. 'ns' indicates no statistical significance. * P < 0.05, ** P < 0.01, *** P < 0.001, **** P < 0.0001. Extended Fig. 7: Densitometric quantification of Western blot analyses presented in Fig. 4G. Quantification of DNA-PKcs、Ku70、Rad51、γ-H2AX levels in A549 and B16 from Fig. 4G. Extended Fig [file 12885_2026_15816_MOESM1_ESM.zip › Extend Fig 8.jpg]

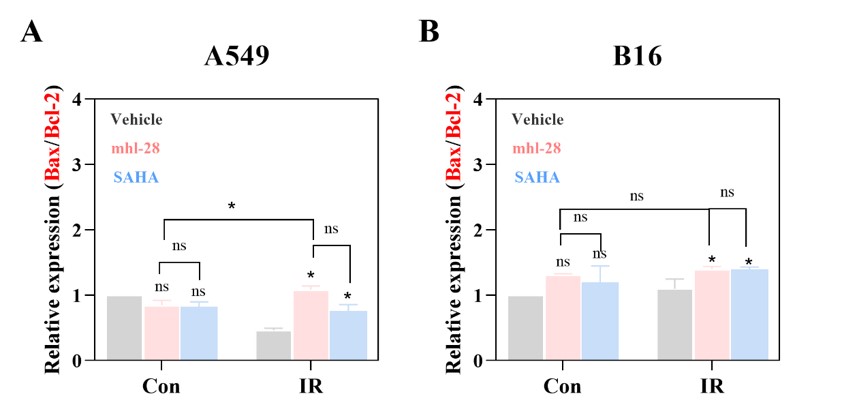

Supplement: Supplementary file 1 — Supplementary Material 1: Extended Fig. 1：Densitometric quantification of Western blot analyses presented in Fig. 1A. Quantification of p-STAT3 (Tyr705) levels normalized to total STAT3 in A549 and B16 from Fig. 1A. Extended Fig. 2：Densitometric quantification of Western blot analyses presented in Fig. 1B. Quantification of Ac-Tub、Ac-α-Tub、Ac-H3/H3、Ac-H4/H4 levels in A549 and B16 from Fig. 1B. Extended Fig. 3：Densitometric quantification of Western blot analyses presented in Fig. 1C. Quantification of p-STAT3 (Tyr705) levels normalized to total STAT3 in A549 and B16 from Fig. 1C. Extended Fig. 4：Densitometric quantification of Western blot analyses presented in Fig. 1D. Quantification of Ac-Tub、Ac-α-Tub、Ac-H3/H3、Ac-H4/H4 levels in A549 and B16 from Fig. 1D. Extended Fig. 5: mhl-28 inhibits tumor cell proliferation. Cell viability in A549 (A), MDA-MB-231 (B) , and B16 (C) cells assessed using CCK8 assay after 48 hours of treatment with different concentration of mhl-28. The vehicle control group was treated with 0.1% DMSO. Data are presented as mean ± SD from three independent experiments (n=3). 'ns' indicates no statistical significance, * P < 0.05, ** P < 0.01, *** P < 0.001, and **** P < 0.0001. Extended Fig. 6: Representative images of colony formation (A) and quantified survival fractions (B) in A549 cells treated with various concentrations of mhl-28 and SAHA. Data are presented as mean ± SD from three independent experiments (n=3). Statistical significance: Asterisks above bars (*, **, ***, ****) indicate comparisons with the consentration-only control group at the same dose. Asterisks above brackets (*, **, ***, ****) indicate pairwise comparisons within the bracketed groups. 'ns' indicates no statistical significance. * P < 0.05, ** P < 0.01, *** P < 0.001, **** P < 0.0001. Extended Fig. 7: Densitometric quantification of Western blot analyses presented in Fig. 4G. Quantification of DNA-PKcs、Ku70、Rad51、γ-H2AX levels in A549 and B16 from Fig. 4G. Extended Fig [file 12885_2026_15816_MOESM1_ESM.zip › Extend Fig 9.jpg]
