# Supplementary material for: The radiosensitizing effects of a STAT3/HDAC dual-target inhibitor derived from isoalantolactone in solid tumor models
Source: BMC Cancer. 2026 Mar 6;26:493. doi: 10.1186/s12885-026-15816-7 (PMC13088848; doi:10.1186/s12885-026-15816-7)
Supplement: Supplementary file 2 — Supplementary Material 2. [file 12885_2026_15816_MOESM2_ESM.zip › Original Images of Blots-mhl-28-with some full-length blots.pptx]

## Slide 1
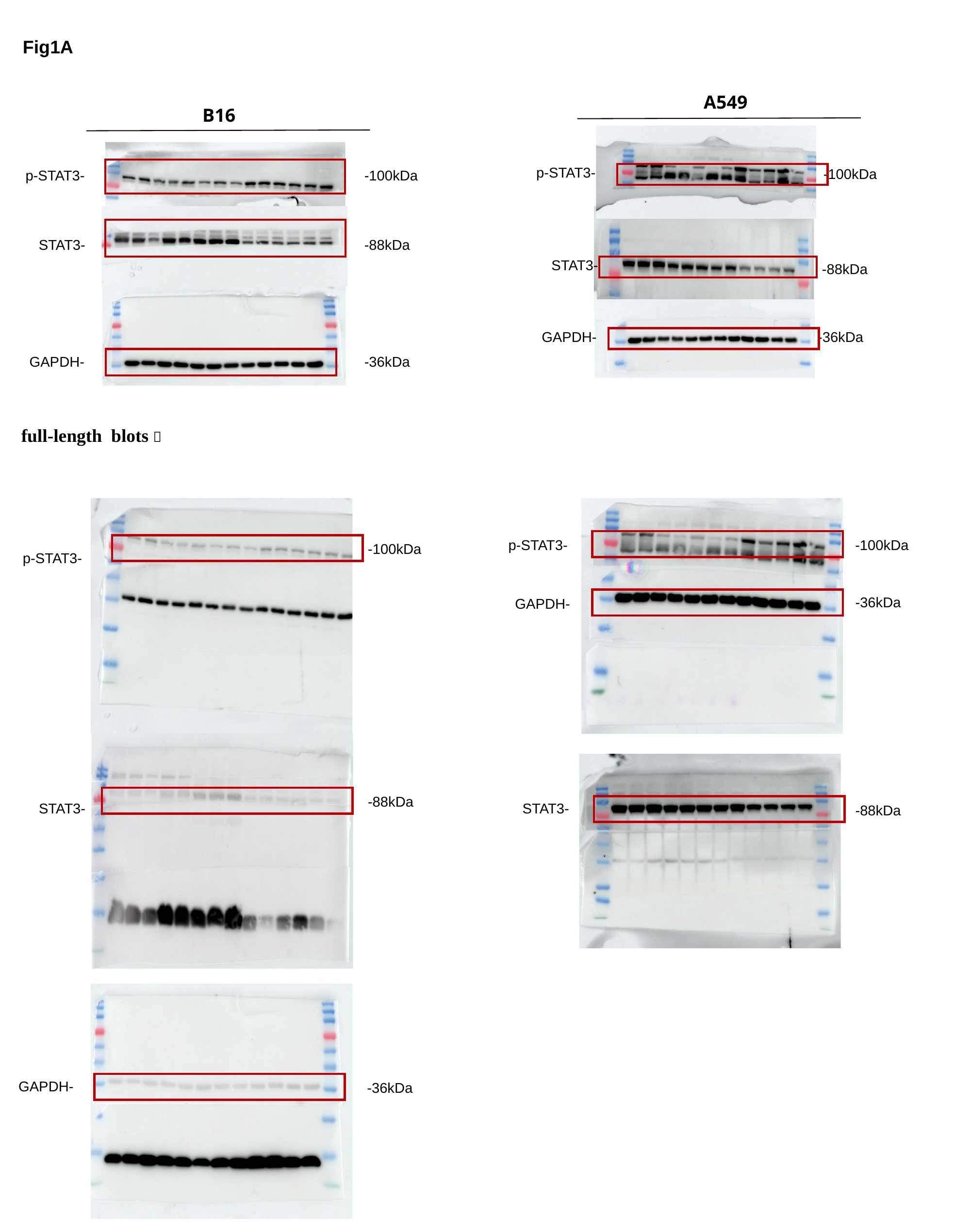

Fig1A
A549
p-STAT3-
-100kDa
STAT3-
-88kDa
GAPDH-
-36kDa
B16
p-STAT3-
-100kDa
STAT3-
-88kDa
GAPDH-
-36kDa
full-length blots：
-100kDa
p-STAT3-
p-STAT3-
-100kDa
-36kDa
GAPDH-
-88kDa
STAT3-
STAT3-
-88kDa
GAPDH-
-36kDa

## Slide 2
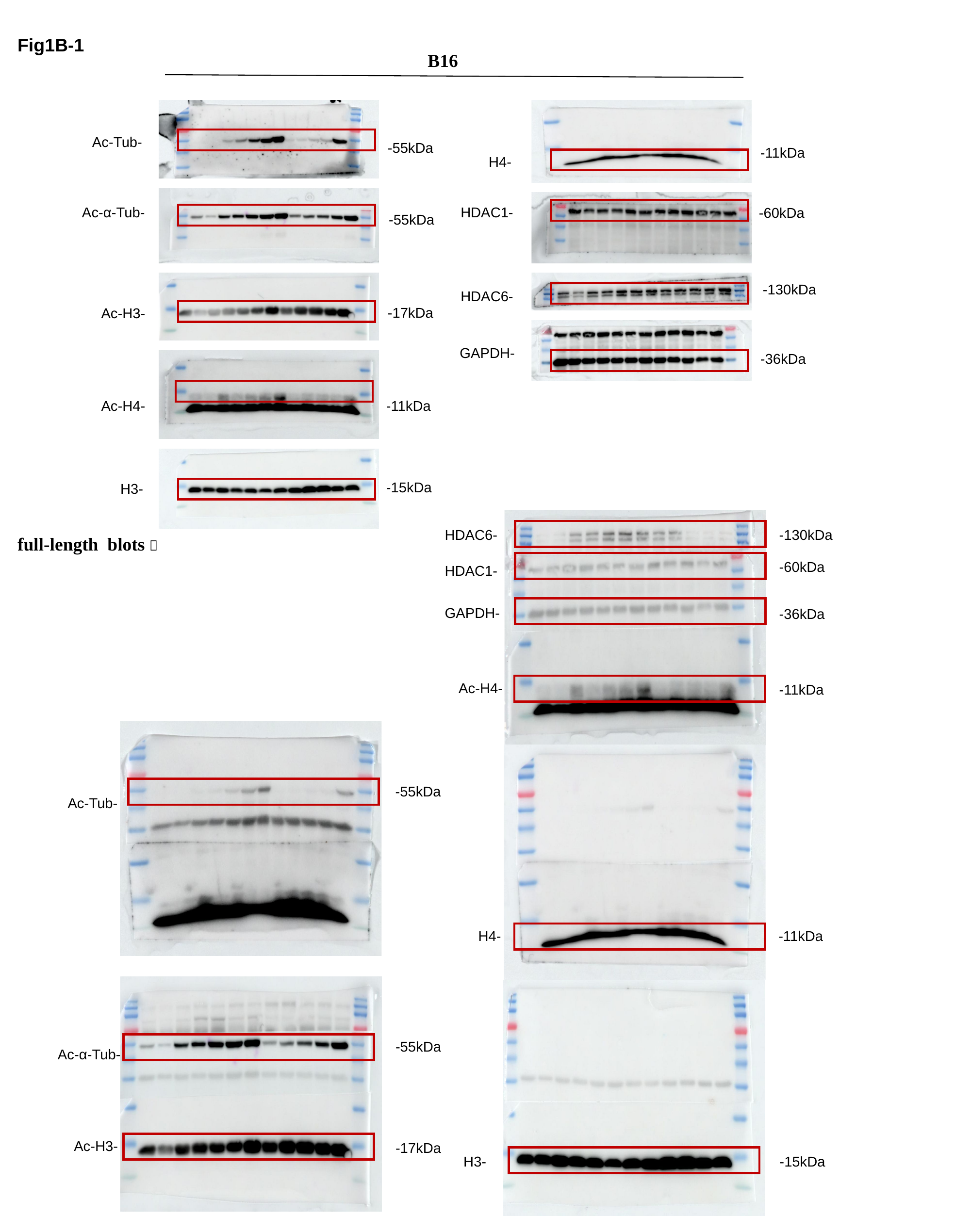

Fig1B-1
B16
-11kDa
H4-
HDAC1-
-60kDa
-130kDa
HDAC6-
GAPDH-
-36kDa
Ac-Tub-
-55kDa
Ac-α-Tub-
-55kDa
-17kDa
Ac-H3-
Ac-H4-
-11kDa
-15kDa
H3-
HDAC6-
-130kDa
-60kDa
HDAC1-
GAPDH-
-36kDa
Ac-H4-
-11kDa
full-length blots：
-55kDa
Ac-Tub-
H4-
-11kDa
-55kDa
Ac-α-Tub-
Ac-H3-
-17kDa
H3-
-15kDa

## Slide 3
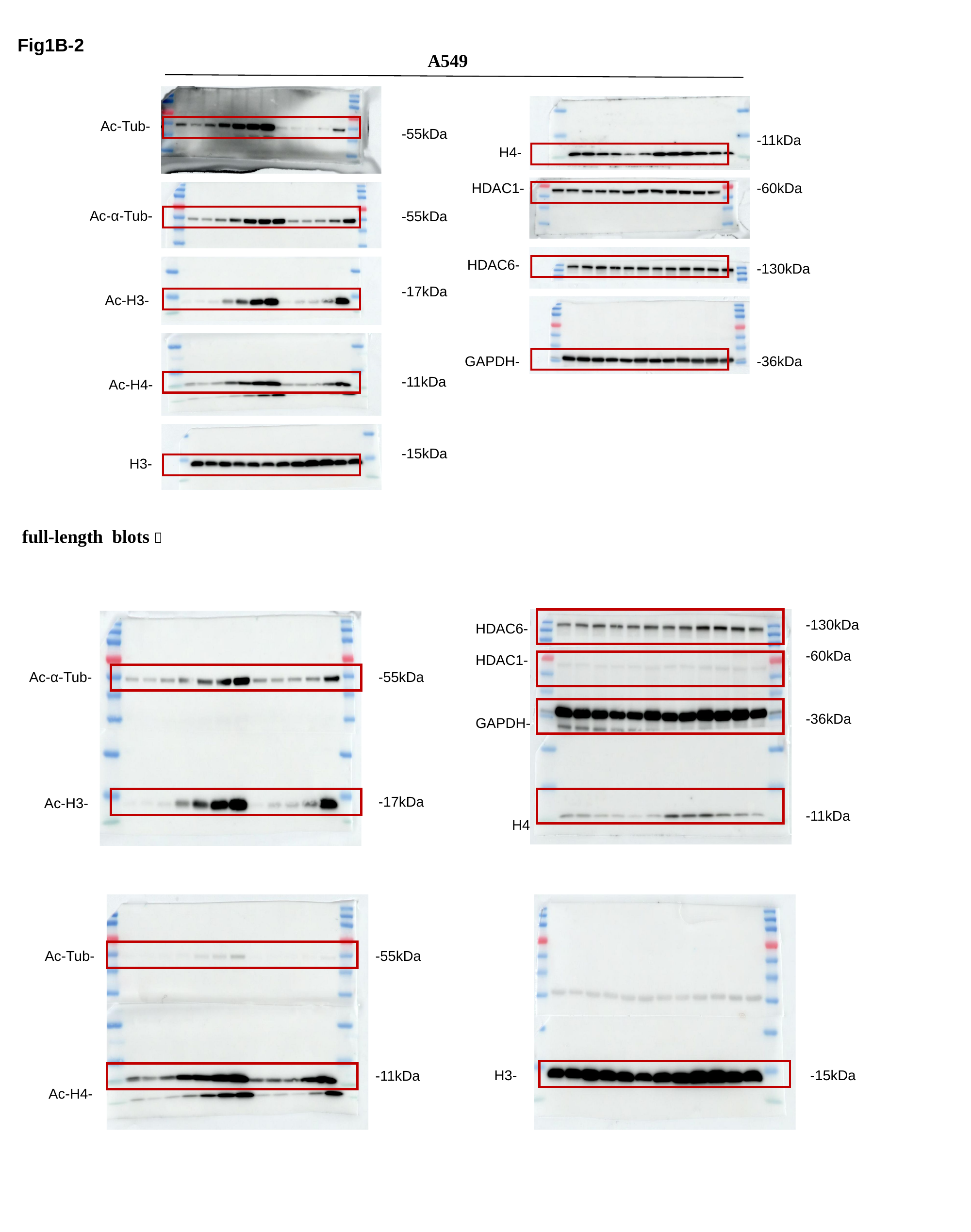

Fig1B-2
A549
Ac-Tub-
Ac-α-Tub-
Ac-H3-
Ac-H4-
H3-
-55kDa
-55kDa
-17kDa
-11kDa
-15kDa
-11kDa
H4-
-60kDa
HDAC1-
HDAC6-
-130kDa
GAPDH-
-36kDa
full-length blots：
-130kDa
HDAC6-
-60kDa
HDAC1-
-36kDa
GAPDH-
-11kDa
H4-
Ac-α-Tub-
-55kDa
-17kDa
Ac-H3-
Ac-Tub-
-55kDa
-11kDa
Ac-H4-
H3-
-15kDa

## Slide 4
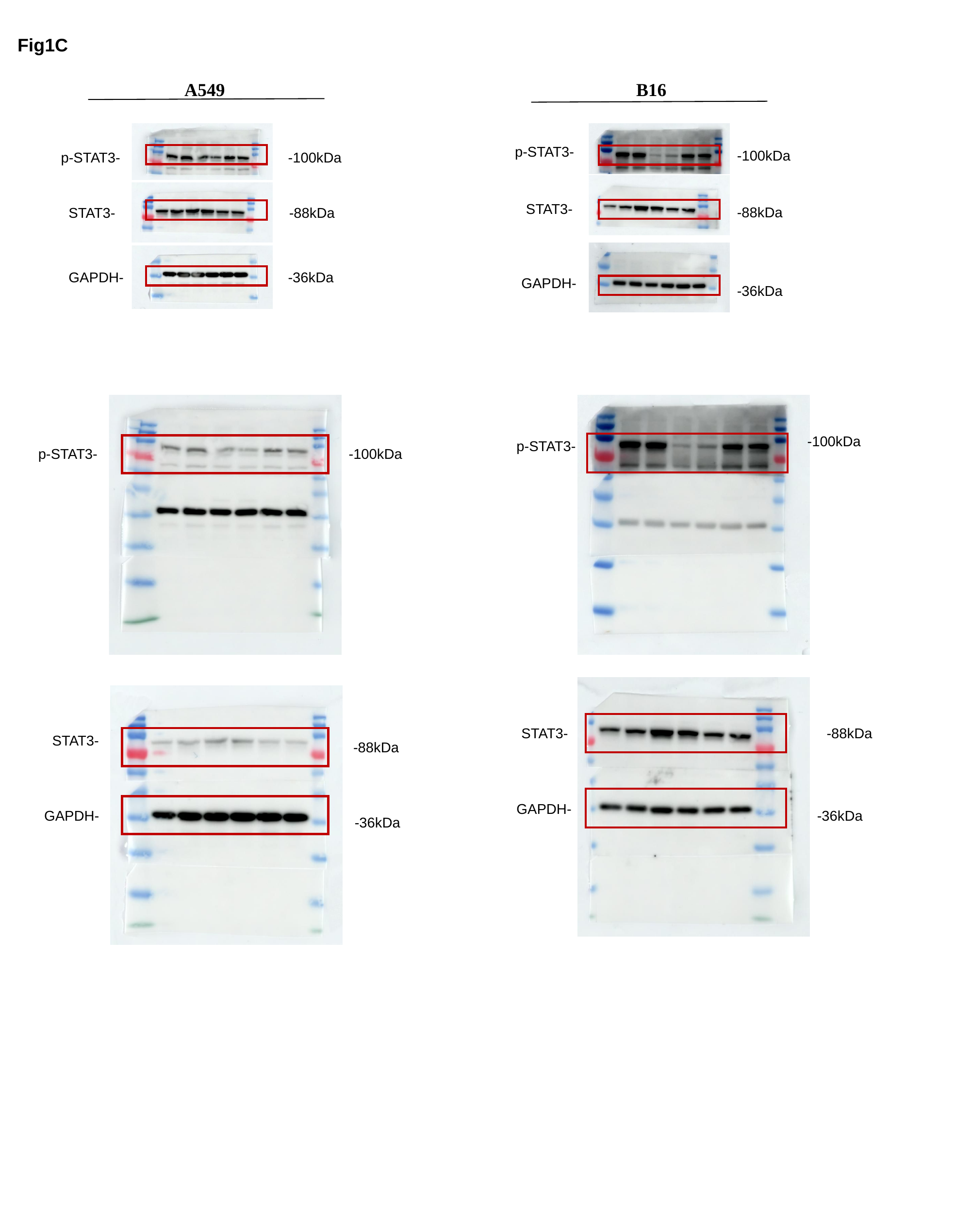

Fig1C
B16
p-STAT3-
-100kDa
STAT3-
-88kDa
GAPDH-
-36kDa
A549
p-STAT3-
-100kDa
STAT3-
-88kDa
GAPDH-
-36kDa
p-STAT3-
-100kDa
-100kDa
p-STAT3-
STAT3-
-88kDa
GAPDH-
-36kDa
STAT3-
-88kDa
GAPDH-
-36kDa

## Slide 5
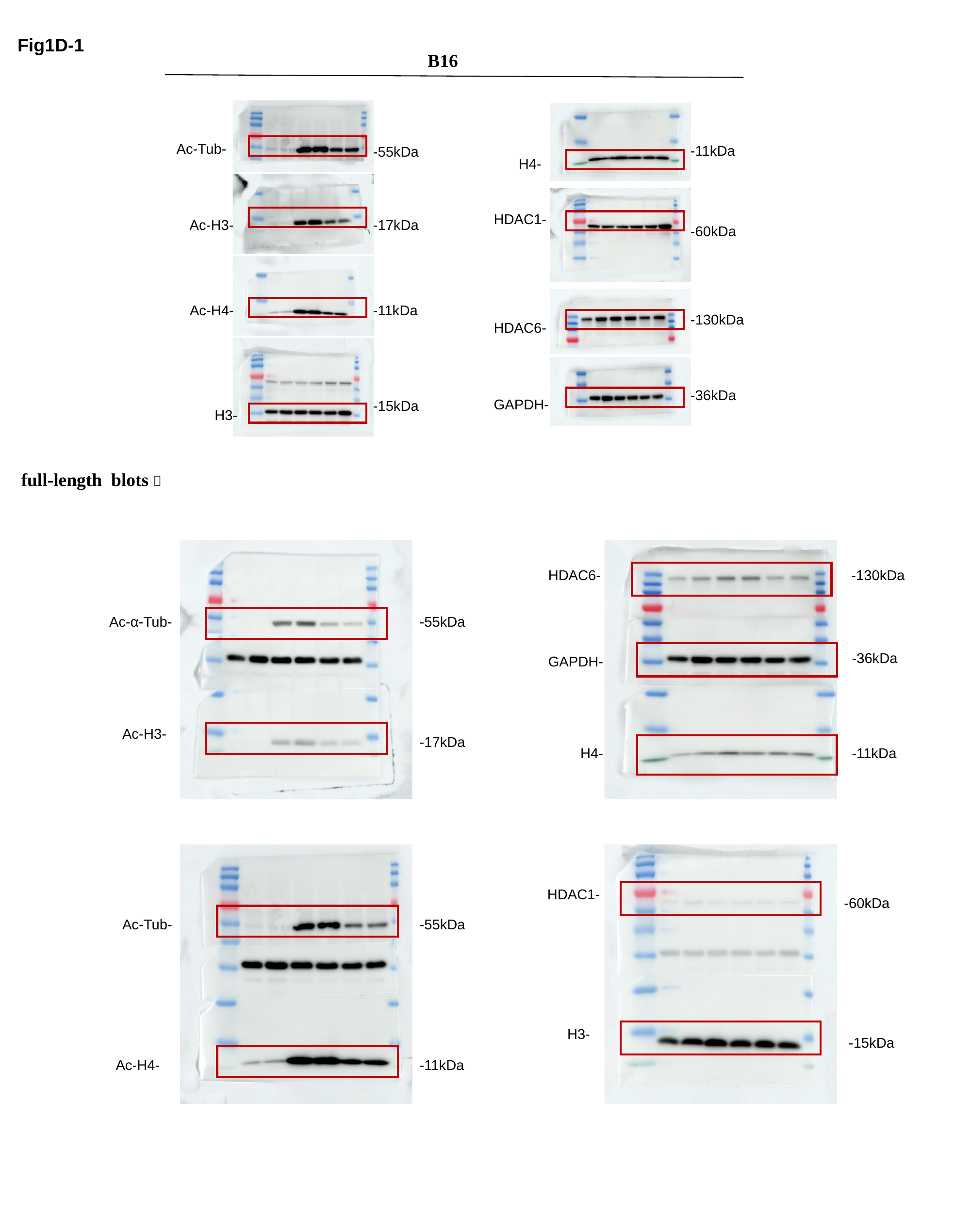

Fig1D-1
B16
Ac-Tub-
-55kDa
Ac-H3-
-17kDa
Ac-H4-
-11kDa
-15kDa
H3-
-11kDa
H4-
HDAC1-
-60kDa
-130kDa
HDAC6-
-36kDa
GAPDH-
full-length blots：
Ac-α-Tub-
-55kDa
Ac-H3-
-17kDa
HDAC6-
-130kDa
-36kDa
GAPDH-
H4-
-11kDa
Ac-Tub-
-55kDa
Ac-H4-
-11kDa
HDAC1-
-60kDa
H3-
-15kDa

## Slide 6
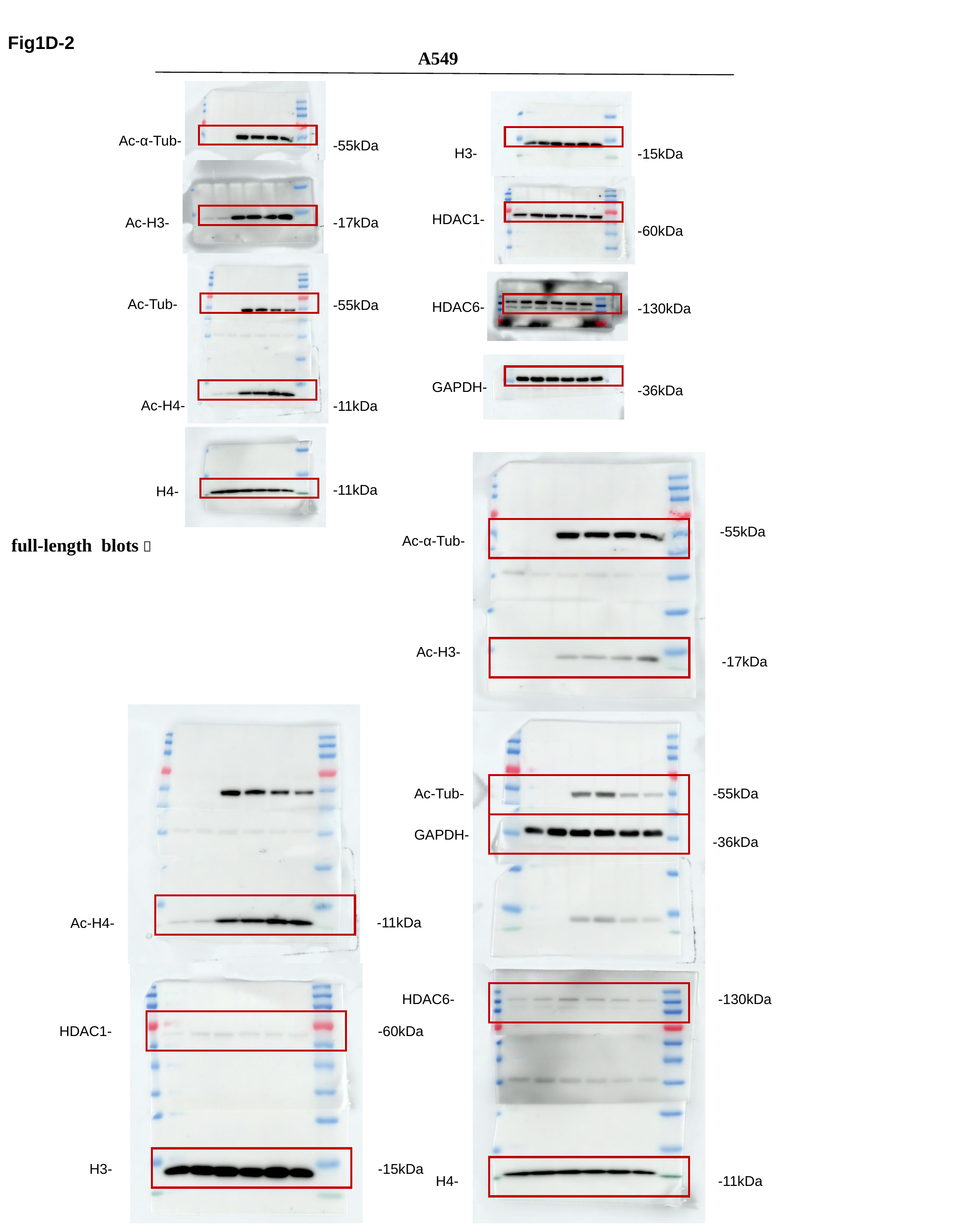

Fig1D-2
A549
Ac-α-Tub-
-55kDa
Ac-H3-
-17kDa
Ac-Tub-
-55kDa
Ac-H4-
-11kDa
-11kDa
H4-
H3-
-15kDa
HDAC1-
-60kDa
HDAC6-
-130kDa
GAPDH-
-36kDa
-55kDa
Ac-α-Tub-
Ac-H3-
-17kDa
full-length blots：
-11kDa
Ac-H4-
Ac-Tub-
-55kDa
GAPDH-
-36kDa
HDAC1-
-60kDa
H3-
-15kDa
HDAC6-
-130kDa
H4-
-11kDa

## Slide 7
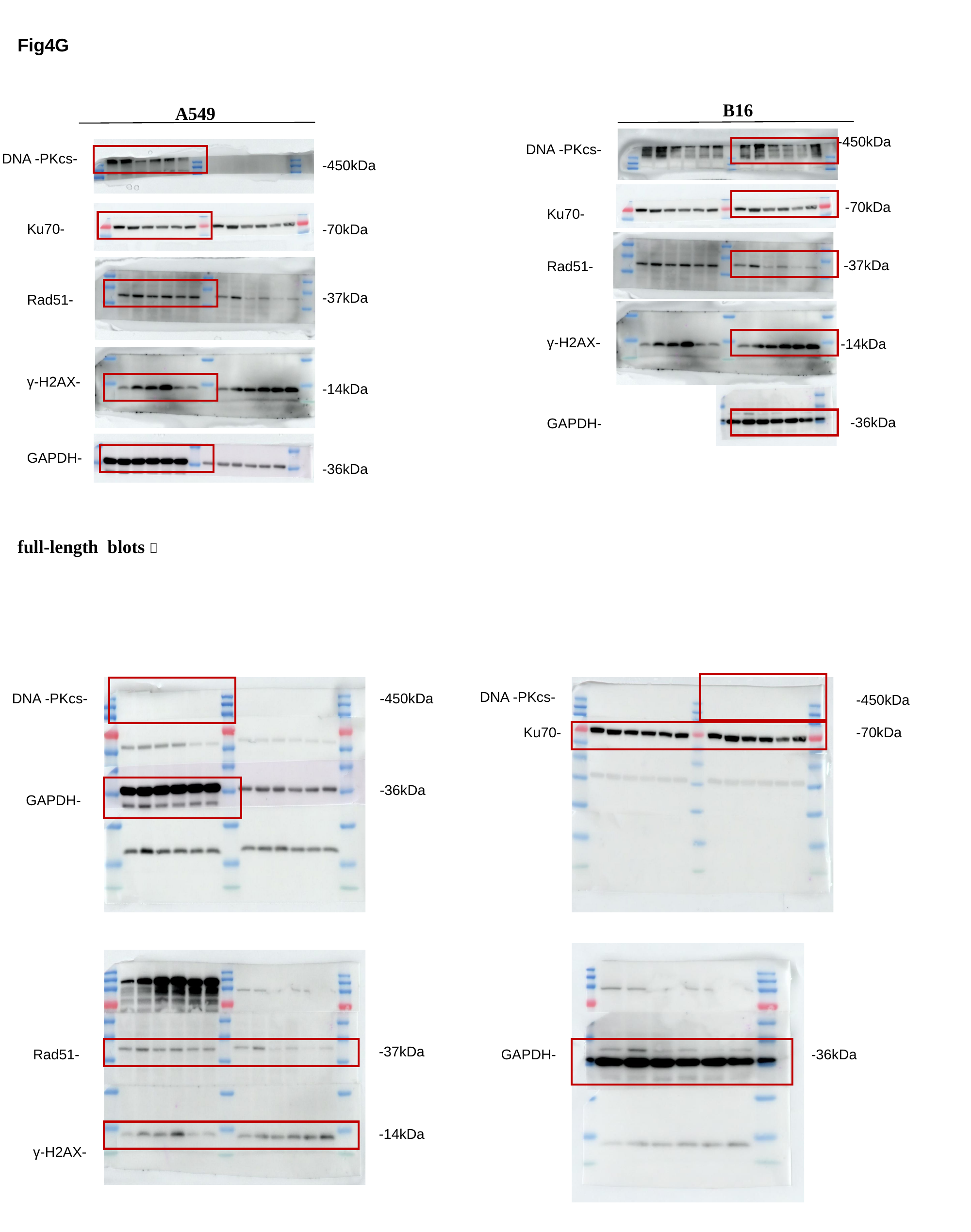

Fig4G
B16
-450kDa
DNA -PKcs-
Ku70-
Rad51-
γ-H2AX-
GAPDH-
-70kDa
-37kDa
-14kDa
-36kDa
A549
-450kDa
Ku70-
-70kDa
-37kDa
Rad51-
γ-H2AX-
-14kDa
GAPDH-
-36kDa
DNA -PKcs-
full-length blots：
Ku70-
-70kDa
DNA -PKcs-
-450kDa
DNA -PKcs-
-450kDa
-36kDa
GAPDH-
-37kDa
Rad51-
-14kDa
γ-H2AX-
GAPDH-
-36kDa

## Slide 8
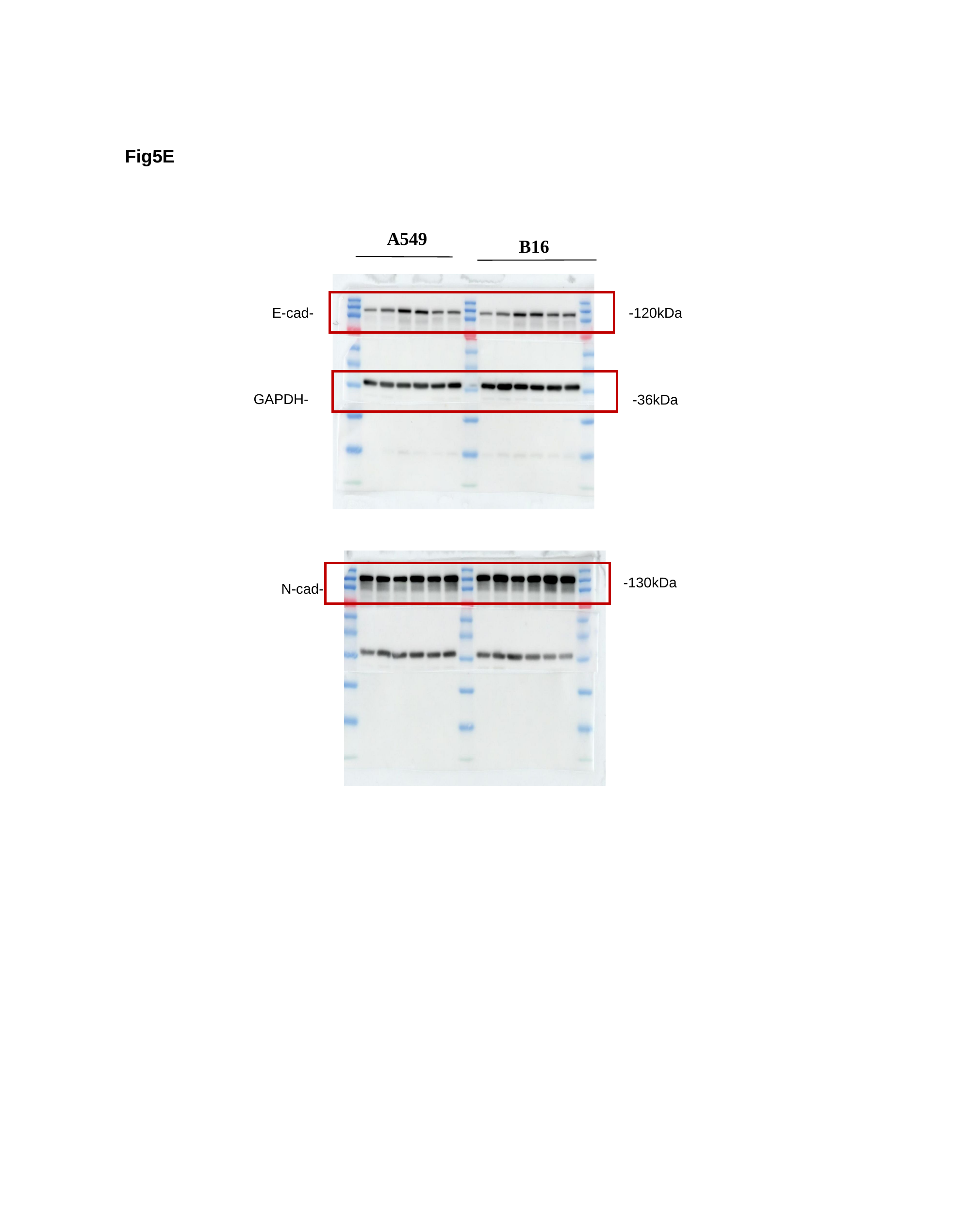

Fig5E
A549
B16
E-cad-
-120kDa
GAPDH-
-36kDa
-130kDa
N-cad-

## Slide 9
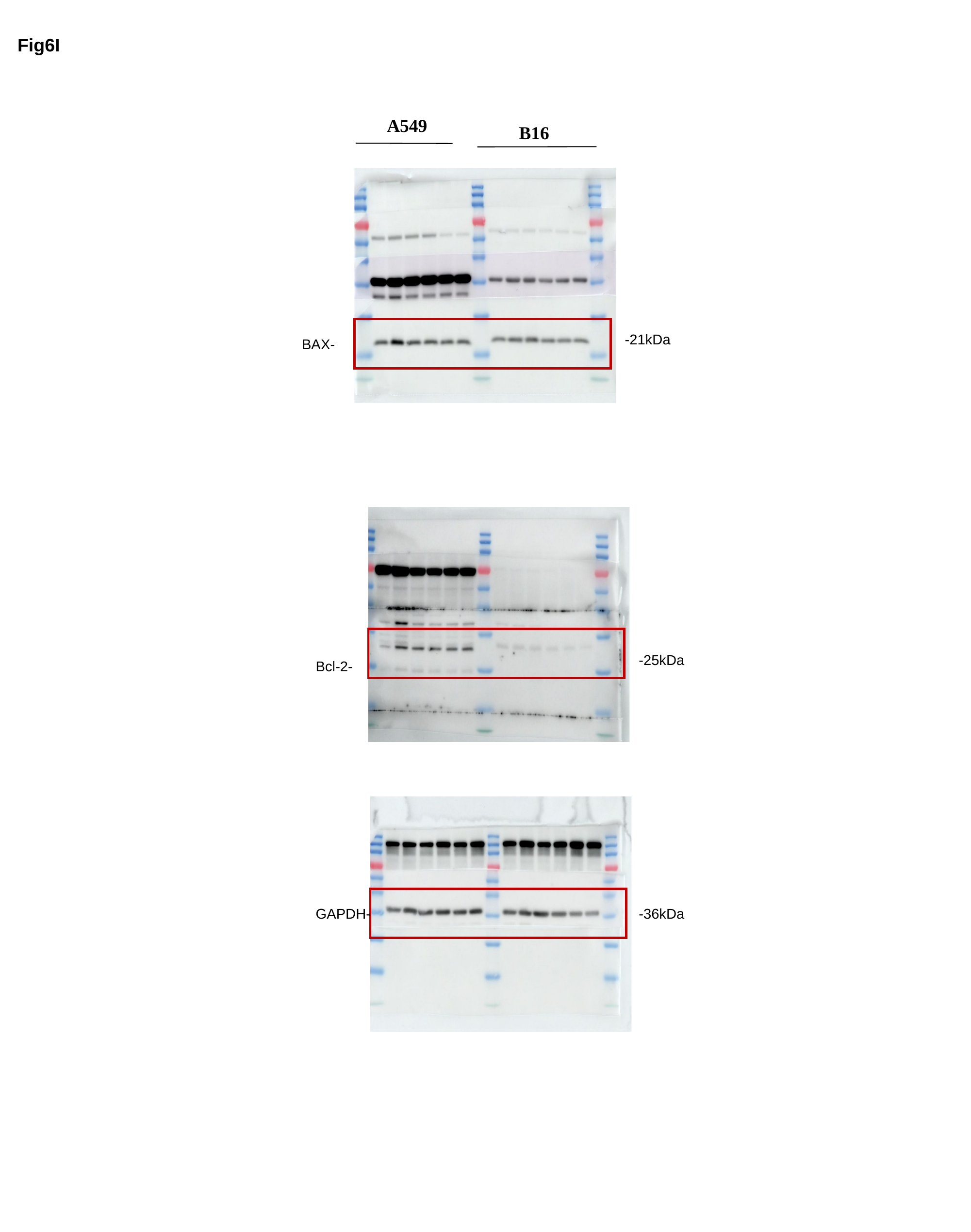

Fig6I
A549
B16
-21kDa
BAX-
-25kDa
Bcl-2-
GAPDH-
-36kDa

## Slide 10
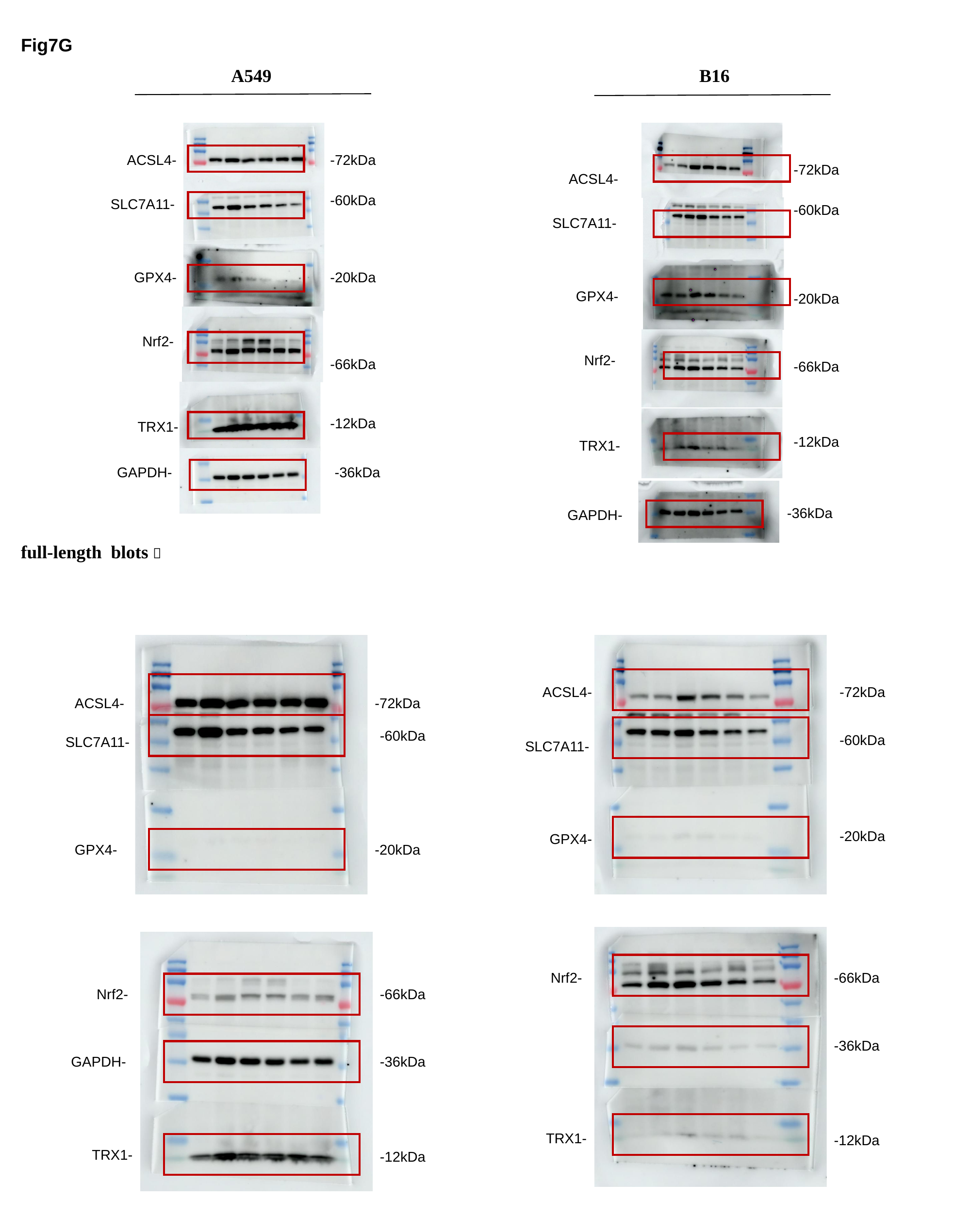

Fig7G
A549
B16
ACSL4-
-72kDa
-60kDa
SLC7A11-
GPX4-
-20kDa
Nrf2-
-66kDa
-12kDa
TRX1-
GAPDH-
-36kDa
-72kDa
ACSL4-
-60kDa
SLC7A11-
GPX4-
-20kDa
Nrf2-
-66kDa
-12kDa
TRX1-
-36kDa
GAPDH-
full-length blots：
ACSL4-
-72kDa
-60kDa
SLC7A11-
GPX4-
-20kDa
ACSL4-
-72kDa
-60kDa
SLC7A11-
-20kDa
GPX4-
Nrf2-
-66kDa
-36kDa
TRX1-
-12kDa
Nrf2-
-66kDa
GAPDH-
-36kDa
TRX1-
-12kDa
